# Taxonomic revision of the *Graphipterus serrator* (Forskål) group (Coleoptera, Carabidae): an increase from five to 15 valid species

**DOI:** 10.3897/zookeys.753.22366

**Published:** 2018-04-26

**Authors:** Ittai Renan, Thorsten Assmann, Amnon Freidberg

**Affiliations:** 1 Department of Zoology, The Steinhardt Museum of Natural History, Tel Aviv University, POB 39040, Tel Aviv, Israel; 2 Institute of Ecology, Leuphana University Lüneburg, Universitätsallee 1, D-21335 Lüneburg, Germany

**Keywords:** Allopatry, conservation status, ground beetles, Harpalinae, Lebiini, species delimitation, sand dunes, sympatry

## Abstract

The south-west Palaearctic *Graphipterus serrator* group is revised. The systematic concept of the *G. serrator* group has undergone many changes during the last two centuries, and several different classifications have been published in recent decades. Here, the numerical taxonomy approach is used with the morphological characterization similarity level of the sympatric taxa in order to delimit allopatrically occurring taxa at the species and subspecies level. A key to the species and distribution maps are provided along with analyses of the conservation status and habitat preferences of the taxa. The *Graphipterus serrator* group currently comprises 16 taxa. Five new species are described: *Graphipterus magnus* Renan & Assmann, **sp. n.**, *Graphipterus mauretensis* Renan & Assmann, **sp. n.**, *Graphipterus piniamitaii* Renan & Freidberg, **sp. n.**, *Graphipterus sharonae* Renan & Assmann, **sp. n.**, and *Graphipterus stagonopsis* Renan & Assmann, **sp. n.** In addition, five taxa are revalidated to full species status: *Graphipterus heydeni* Kraatz, 1890, **stat. rest.** (lectotype designated), *Graphipterus multiguttatus* (Olivier, 1790), **stat. rest.** (lectotype designated), *Graphipterus peletieri* Laporte de Castelnau, 1840, **stat. rest.** (the frequently used name *lepeletieri* is an error), *Graphipterus rotundatus* Klug, 1832, **stat. rest.** (lectotype designated), and *Graphipterus valdanii* Guérin-Méneville, 1859 **stat. rest.**, and a full species status is proposed for *Graphipterus reymondi* Antoine, 1953, **stat. n.** One new synonymy is proposed: *Graphipterus kindermanni* Chaudoir, 1871, **syn. n**. of *Carabus multiguttatus* Olivier, 1790. Lectotype designations were made for *Graphipterus heydeni*, *Graphipterus minutus* Dejean, 1822, *Graphipterus multiguttatus*, and *Graphipterus rotundatus*. Neotype designations were made for *Graphipterus reichei* Guérin-Méneville, 1859, *Graphipterus intermedius* Guérin-Méneville, 1859, and *Graphipterus valdanii* Guérin-Méneville, 1859.

## Introduction

The ground beetles (Carabidae) constitute one of the largest animal families. They include almost 40,000 described species, distributed throughout every continent (Lorenz 2005). Harpalinae Bonelli, 1810 comprise one of the largest subfamilies of the Carabidae, whose taxonomy is poorly known due to the lack of modern revisions for most of its genera and tribes (Erwin et al. 2012). The subtribe Graphipterina Latreille, 1802 belongs to the tribe Lebiini Bonelli, 1810, which has still not been satisfactorily resolved phylogenetically (Ober and Maddison 2008) and is one of the largest tribes of the given subfamily. The nominate genus *Graphipterus* Latreille, 1802 has been previously revised four times: Chaudoir (1870); Péringuey (1896), focusing on South Africa fauna; Burgeon (1929); Basilewsky (1977). The last revision includes 116 species distributed throughout Africa except for the central Sahara desert and the tropical forest regions. Since then, only few taxonomic studies have been published (Basilewsky 1981, 1986; Werner 2003, 2007; Mawdsley 2012), including the extensive systematic and taxonomic overview of the Carabidae of the World (Lorenz 2005).

The members of the *Graphipterus serrator* (Forskål, 1775) group differ from most of the other 138 *Graphipterus* species (Lorenz 2005, Werner 2007, Mawdsley 2012) by their unique distribution. Together with *Graphipterus exclamationis* (Fabricius 1792), they are the only taxa of this genus that are distributed in and north of the Sahara. All other *Graphipterus* species have distribution ranges restricted to the arid and sub-tropical regions of central and southern Africa.

The systematic concept of the *G. serrator* group had undergone changes many times during the last 200 years, and numerous taxonomic publications have dealt with members of this group (e.g., Olivier 1790, Dejean 1822, Guérin-Méneville 1859, Klug 1832, Chaudoir 1870, Kraatz 1890, Péringuey 1896, Burgeon 1929, Schatzmayr 1936). During the last 40 years, several influential taxonomic publications have presented very different species classification of the *G. serrator* group (Alfieri 1976, Basilewsky 1977, Hůrka, 2003, Lorenz 2005). Basilewsky (1977), as part of his broad scope revision, recognized two species in the *G. serrator* group: *G. serrator* (Forskål, 1775), with six subspecies; and *G. minutus*, Dejean 1822, with two subspecies. However, Basilewsky’s species concept strongly tended towards the “lumping” approach of taxonomy (Dayrat 2005). By choosing to re-rank six subspecies under one species, Basilewsky ignored two basic criteria that were already well accepted at his time: 1. Two or more subspecies of the same species cannot co-occur in sympatry in one location or well defined ecological habitat. 2. Subspecies are expected to share the same dominant characters (Mayr 1969). However, each of the *G. serrator* subspecies in the sense of Basilewsky does co-occur with at least one other subspecies, and they are usually characterized by different shapes of the median lobe of the aedeagus. Consequently, some modern authors have accepted at least some of “Basilewsky’s subspecies” as “good” species, even though they do not recognize all co-occurring taxa of the group as species. However, Lorenz (2005) and Huber and Marggi (2017), are identical by the taxonomic ranking of taxa within the *G. serrator* group: five species, four of them polytypic ones (Huber and Marggi 2017).

Only a rigorous morphological revision of the *Graphipterus serrator* group with a critical analysis of previous classifications can solve the problems resulting from these diverging classifications. Furthermore, an approach to define a threshold for species delimitation from sympatric taxa is needed in order to cope with the general problem of treating allopatric taxa as species or subspecies.

Several species concepts are known in modern taxonomy and systematics (e.g., Claridge et al. 1997, Zachos 2016). Some of these are controversial, with potentially serious effect on the conservation of species or other biodiversity elements (May 1990). As an approach that tends to reduce the probability of overlooking species was used, an increased number of species was found in the *Graphipterus serrator* group, indicating that at least some populations and taxa require proper conservation efforts in order to ensure their long-term survival. The *Graphipterus serrator* group comprises terrestrial wingless beetles, with a highly specific habitat preference and usually distributed over limited geographical ranges. Some members of this group inhabit coastal regions of the Atlantic Ocean and the Mediterranean Sea, and thus belong to the most threatened regions in the world (Samways 1994, Brooks et al. 2002, Cuttelod et al. 2008). Consequently, the *G. serrator* group constitutes a special object for conservation efforts. Here, the new understanding of the classification and distribution patterns within the *G. serrator* species group are employed as well as ecological and conservation biological information summarized to provide the first analysis of the conservation status of these taxa.

From a general biological point of view, a taxonomic revision of the given group is needed, as numerous aspects of its biology, ecology, and morphology have already been studied. *Graphipterus serrator* (*sensu lato*) is one of the most conspicuous and familiar ground beetles in the Palaearctic region. Unusually among beetles, *G. serrator* has been the subject of many studies dealing with a wide range of topics: larval morphology (Brandmayr et al. 1993, Brandmayr et al. 1994a), adult morphology (Pocock 1902), adult anatomy (Bugnion 1933), adult and larval ecology (Paarmann 1985, Paarmann et al. 1986, Brandmayr et al. 1994b, Dinter et al. 2002), and genetics (Wahrman 1966).

The aim of the current work is to revise the south-west Palaearctic *Graphipterus serrator* group, based on objective species delimitation. The monograph presents re-descriptions of eleven taxa with a new status and five new species. Moreover, an updated identification key and distribution maps for all species of the group are provided.

## Materials and methods

More than 4,000 specimens were examined for this study, including all available holotypes, syntypes, and paratypes. The material is stored in the following collections:

**AVTC** Augusto Vigna Taglianti, Rome, Italy, private collection

**BMNH** The Natural History Museum, London, United Kingdom

**CAB** Working collection Thorsten Assmann, Bleckede, Germany (part of ZSM)

**CAMMZ** Cambridge University Museum of Zoology, Cambridge, United Kingdom

**DWC** Working collection D.W. Wrase, Berlin, Germany (part of ZSM)

**KCE** Kibbutzim College of education, Tel Aviv, Israel

**NBC** Naturalist Biodiversity Center, Leiden, The Netherlands

**NHMB** Naturhistorisches Museum Basel, Switzerland

**NHMB** Entomology Department, Muséum National d'Histoire Naturelle, Paris, France

**NMP** The National Museum, Prague, Czech Republic

**RMRAC** Royal Museum for Central Africa, Tervuren, Belgium

**SDEI** Senckenberg German Entomological Institute Müncheberg (= Senckenberg Deutsches Entomologisches Institut Müncheberg), Germany

**SMNHTAU** Steinhardt Museum for Natural History, Tel Aviv University, Tel Aviv, Israel

**ZMHB** Zoologisches Museum, Humboldt Universität, Berlin, Germany

**ZMK** Zoological Museum, University of Kiel, Kiel, Germany

**ZMUC** Natural Museum of Denmark, Zoological Museum, Copenhagen, Denmark

**ZSM** Zoological State Collection Munich, Munich, Germany

*Images*: Macrophotographs were taken with a Leica M205 C Stereomicroscope, FusionOptics – Objective Planapo 0.63× M-Serie in combination with a Leica DMC4500 digital camera LAS Montage MultiFocus. The habitus photographs were taken with a Canon D65 and the objective Canon MP-E 65 mm.

*Measurements*: All measurements were made with an ocular micrometer on a Leica M80 stereomicroscope. When possible, the largest, smallest and three medium sized intact specimens of both sexes for each species were chosen for measurements which include:

**BL** Body length

**BPW** Basal pronotum width (minimum pronotum width)

**EL** Elytra length (from apical point of scutellum to apex)

**EL/EW** Elytra length/width

**EW** Maximum elytra width

**EYL** Eye length

**HW** Width of head

**HW/PW** Head/pronotum width

**MTAL** Metatarsus length

**MTIL** Metatibia length

**PL** Pronotum length (from apex to base along median impression)

**PL/PW** Pronotum length/width

**Figure 1.**
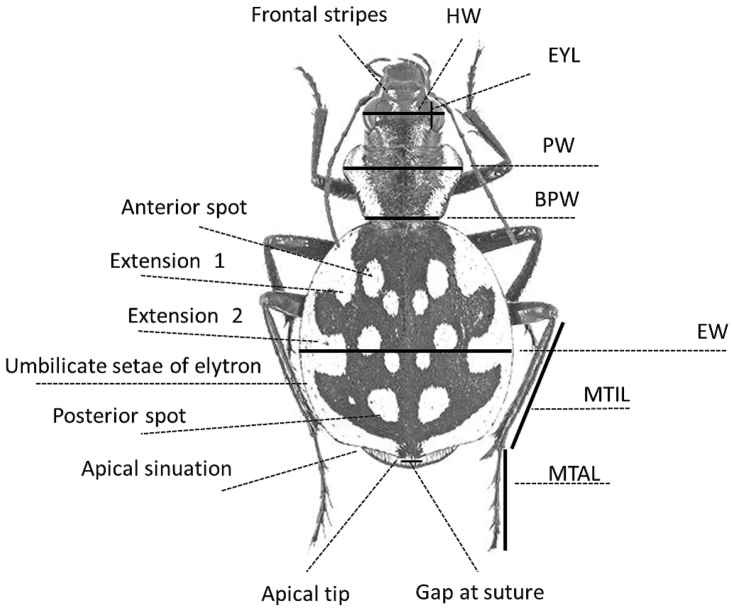
Patterns and morphological characters used in the descriptions (*Graphipterus serrator*).

**PW** Maximum pronotum width (Fig. 1)

Whenever in the text the words large, medium or small size appear, they are in comparison to the average of the other group species: Length, BL: small (11.9–13.5), medium (14–17.1), large (17.4–19.5); Head, HW/PW: slender (0.70–0.739), medium (0.74–0.79), wide (0.78–0.84); Legs, El/MTIL: short (1.60–1.89), medium (1.64–1.74), long (1.53–1.58). All comparative elements of the descriptions mean relative to the other species of the *Graphipterus serrator* group.

*Acronyms and signs in the material examined*: Aedeagus extracted (ae), unclear (uc), recent names of locations with a valid name are given in square brackets [] in addition to the original names. Original label text of type specimens appears between the symbols < >.

*Scraping record*: In order to examine more characters, the scraping sounds of representative males of *G. serrator* and *G. multiguttatus* were recorded and compared. We used an ultrasonic condenser microphone and a PCTape recording system (custom-made by Tübingen University) under lab conditions.

*Comparison*: In the comparison paragraph for each species, mainly the easily recognizable morphological characters of similar species are provided.

*Distribution data*: The recorded data from the species’ distribution range are collected from approximately 1,400 specimen labels stored in museums and private collections (see above).

*Habitat*: Data on the habitats of the species are derived from surveys by the authors and colleagues.

*Conservation*: Threat assessments for the species are based on the distribution range of each species and the known threats to its habitat. Information about distribution ranges are given following IUCN rules (IUCN 2017).

*Species delimitation*: The species delimitation of the *Graphipterus serrator* group is a substantial challenge due to the more than 200 years of studies by many taxonomists, the rarity of some species, and the limited knowledge on the distribution of many group members. Our study is based on the Biological Species Concept (BSC) following Jordan (1905) and Mayr (1969), and, considering the weakness of this concept (e.g., Meier 2000), a numerical approach for species delimitation is suggested.

Numerical approaches in taxonomy date back to early taxonomic authors, but have been established mainly by Sneath and Sokal (1973). In the consecutive decades, a substantial part of taxonomic studies bases on numerical approaches as they provide objective data. It is still, also in the era of DNA approaches a useful way to delineate taxa (Sneath 1995, Jensen 2009). In ground beetles, numerical approaches are frequently used (e.g., Liebherr 1986, Baehr 1998, Liebherr and Schmidt 2004). Most of them do not have a phenetic, but a phylogenetic basis. However, a phenetic approach over a phylogenetic one is preferred as for almost all characters (e.g., body length, coloration patterns) it is not possible to polarize primitive or derived character states, even by using first and second order outgroups.

An important element of our approach is that to use sympatric taxa to determine the threshold to delineate allopatrically distributed taxa. Of course, this approach may be criticized from the point of view of other species concepts or the phylogeny. Even when we recognize that species delimitations and species limits are in many cases inherently arbitrary, the chosen approach can be applied widely in most species-rich taxa which are at least partly distributed in sympatry. Moreover, the delivery of taxonomic ranking has a high level of objectivity, consistency, and transparency (Tobias et al. 2010). To avoid both taxonomic inflation and “species” with excessive gene flow (cf. Cotterill et al. 2014), numerous authors argue for such an approach, also in recent publications (Tobias et al. 2010, Zachos et al 2013, Assmann et al. 2008).

The cases of the co-occurring taxa in the *Graphipterus serrator* group offer the option to use sympatrically distributed taxa as a reference for the extent of morphological differentiation among species. Criteria and thresholds based on the morphological characterization similarity level of the sympatric taxa are used, in order to apply them to the allopatric taxa to delimit species and subspecies.

The extreme rarity of hybrid specimens of co-occurring species supports our delimitation approach of a threshold based on a characterization similarity level of sympatric taxa. Thousands of specimens in collections and in the field were studied, but only one specimen recognized as a hybrid of *multiguttatus* and *serrator* which co-occur in the northern Negev.

In order to establish quantitative species delimitation, the threshold for delimitation according to the minimum sum of the ‘diagnostic characters’ of the sympatric species pairs was determined. A set of 39 characters (25 morphological and 14 ratios characters) and a matrix of all 120 pair-wise taxon comparisons was used. A diagnostic character constitutes a clear and consistent describable appearance as color pattern or aedeagus shape, or organ ratios. A quantitative measure as a diagnostic character was considered only if it showed a maximum of 5% overlapping between two taxa, or no overlapping at all. Elytral pattern and coloration are generally not well accepted as a character state by which to separate species. However, in *Graphipterus* they mirror many other characters in these states and several recent publications have based their findings mainly on those characters (e.g., Werner 2007; Mawdsley 2012). In this study, these characters were consider along with morphological shapes and measurements. Following Sneath and Sokal (1973), we decided not to give a different “weight” to a given character, as there is no objective way in which to do so.

## Results

### Species delimitation

**Figure 2.**
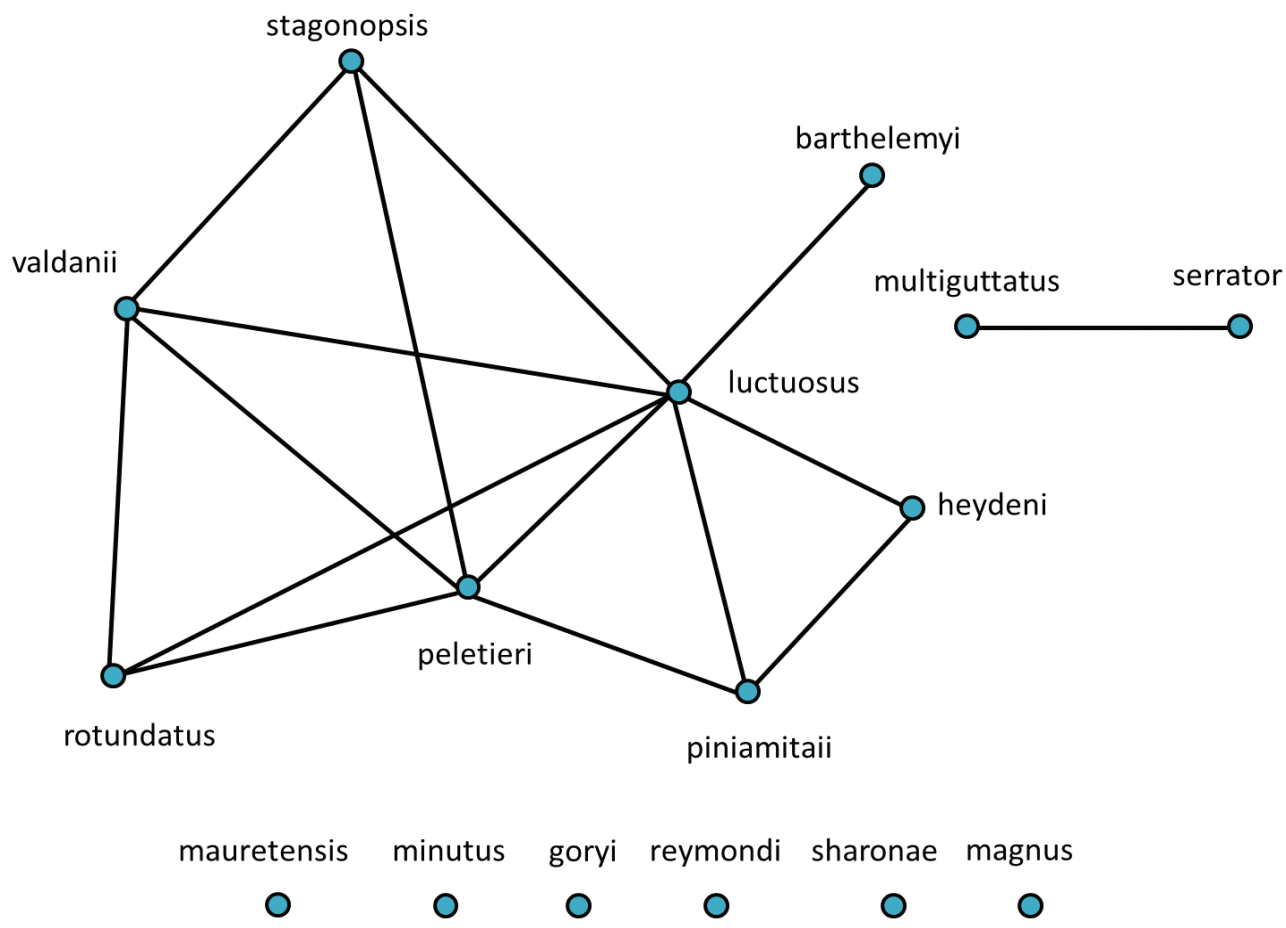
The ten taxa of the *Graphipterus serrator* group that occur sympatrically. Lines connect those taxa that co-occur sympatrically. Bottom row: the exclusively allopatrically occurring species.

Altogether, the matrix of diagnostic characters presents 120 comparison pairs with ten species living in 15 sympatric situations (Fig. 2). The number of diagnostic characters of the sympatric species pairs ranges from six to 18 (Table 1). The sympatric taxon pair *luctuosus* – *peletieri* shows the lowest value (six diagnostic characters) and therefore six was set as the threshold for the ranking of two taxa as “good” species. The allopatric taxon pair *valdanii* – *serrator* differs by six diagnostic characters. Transferring this threshold to the allopatric taxon pairs necessitated our classifying both taxa as “good” species (Fig. 2 and Table 1).

**Table 1.** Matrix of the sum of diagnostic characters for species delimitation. Bold marked are sympatric taxon pairs.

	*serrator*	*barthelemyi*	*heydeni*	*luctuosus*	*magnus*	*mauretensis*	*minutus*	*goryi*	*multiguttatus*	*peletieri*	*piniamitaii*	*reymondi*	*rotundatus*	*sharonae*	*stagonopsis*	*valdanii*
***serrator***		17	10	19	19	15	19	17	**11**	18	18	17	14	13	13	6
***barthelemyi***			15	**17**	15	16	15	14	14	9	14	18	14	13	11	15
***heydeni***				**14**	16	12	16	15	15	11	**14**	14	12	12	11	10
***luctuosus***					12	11	16	13	20	**6**	**8**	12	**14**	14	**13**	**18**
***magnus***						14	19	17	12	16	10	13	8	12	16	19
***mauretensis***							19	17	10	13	12	9	8	9	12	13
***minutus***								4	16	14	16	17	22	20	19	18
***goryi***									22	15	17	16	19	18	17	17
***multiguttatus***										17	13	15	8	7	12	15
***peletieri***											**9**	13	**14**	15	**14**	**16**
***piniamitaii***												15	10	13	12	16
***reymondi***													12	14	12	18
***rotundatus***														9	9	**11**
***sharonae***															10	13
***stagonopsis***																**10**
***valdanii***																

The leading sympatric taxon example is *G. serrator* and *G. multiguttatus* (eleven diagnostic characters) which co-occur in the Sinai Peninsula, Egypt, and the western Negev sand dunes in Israel. The main distinguishing morphological characters between them are: number and pattern of elytral spots and extensions, suture distinctness, elytra cross section shape, colors of spurs and claws, apex pattern and shape of median lobe of aedeagus. Moreover, we know from intensive earlier studies that the habitat preferences of the two species differ from one another (Renan in prep.). This finding provides ecological evidence for a classification based on the morphology as two “good” species.

Taking all taxa into account, the number of diagnostic characters for the pairwise comparisons ranges between four and 21. A value below the threshold of six was found only for the allopatric pair *minutus* and *goryi* and these taxa were treated as subspecies according to our species delimitation. The subspecies classification is in agreement with most other authors (e.g., Hůrka 2003, Lorenz 2005).

All other taxa that show lower values of the diagnostic characters than six (cf. Table 1) were carefully examined for further deviating characters. In none of these cases were any taxonomically useful diagnostic characters found and therefore all these taxa were ranked as junior synonyms (see next chapter).

### Taxonomy

#### Graphipterus

Taxon classificationAnimaliaColeopteraCarabidae

Latreille, 1802

Stagonopterus Chaudoir, 1871 (type species: Carabus serrator Forskål, 1775)
Graphopterus Agassiz, 1847: 167

##### Type species.

*Carabus variegatus* Fabricius, 1792 (= *Carabus serrator* Forskål, 1775).

##### Diagnosis.

The *Graphipterus serrator* group is included in the genus *Graphipterus* based on the following combination of characters:

Clypeus concave at anterior margin, posteriorly well separated from front; labrum wide and short, with well-developed microsculpture and six setiferous pores. Mandibles broad at the base, sharp and strongly curved at tip; labial and maxillary palps long and slender, glabrous with exception of distal end of segments which bear a few hairs; last palpal segments slightly thicker than penultimate ones.

Pronotum transverse and cordiform, slightly convex, usually ornamented by colored scales at the lateral bead, disc with or without scales. Anterior and posterior angles obtuse.

Scutellum triangular, small and short, often hidden by the pronotal base. Flightless. Elytra wide and oval, slightly convex, coalesced along suture, humeri completely rounded; surface covered by dense or sparse scales, white scales creating longitudinal stripes on the radial field and spots on the disc; apex almost truncate. Pygidium not covered by elytra, last visible tergite with colored scales.

Legs long, usually black or brown, protibia with clypsetae (antenna cleaner) and dark parallel spurs, as long as ¾ of protarsomere 1. Mesotibia with two long and thin not serrated spurs, metatibia with one long and thin not serrated spur and one shorter, wide and obtuse spur. Claws of all legs long and smooth on median margin.

### *Graphipterus serrator* group

**Figure 3.**
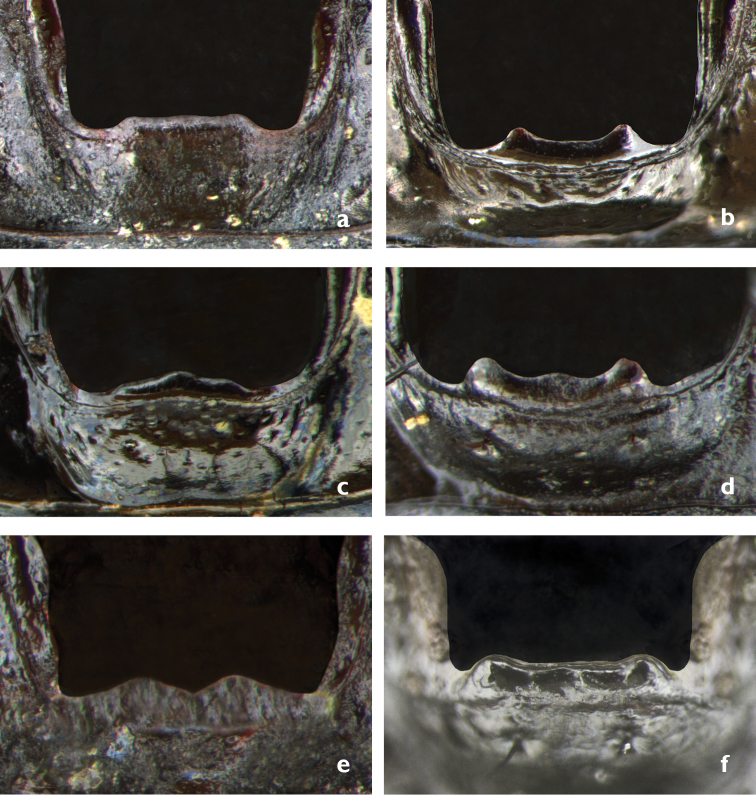
Mentum morphs of the *Graphipterus serrator* group: **a** No teeth (*G. barthelemyi*) **b** Two teeth with concavity between them (*G. heydeni*) **c** Two teeth as margin between them slightly convex in middle. (*G. valdanii*) **d** Three teeth (*G. peletieri*) **e** Two pronounced teeth (*G. minutus minutus*) **f** Three teeth, mid tooth very shallow (*G. serrator*).

Within the genus, the *G. serrator* group is characterized by a combination of the following characters: Antennae reaching elytral humeri; antennomere I wide and glabrous, at apex with two black erect setae; antennomere II half as wide as long and half as wide as antenonmer I, glabrous, at apex with one black erect setae; antennomere III glabrous and four times as long as antennomere II; antennomere IV pubescent in the apical two-thirds; antennomeres V–XI fully pubescent. Mentum without or with one, two, or three teeth, with or without depressions between the teeth (Fig. 3a–f).

**Figure 4.**
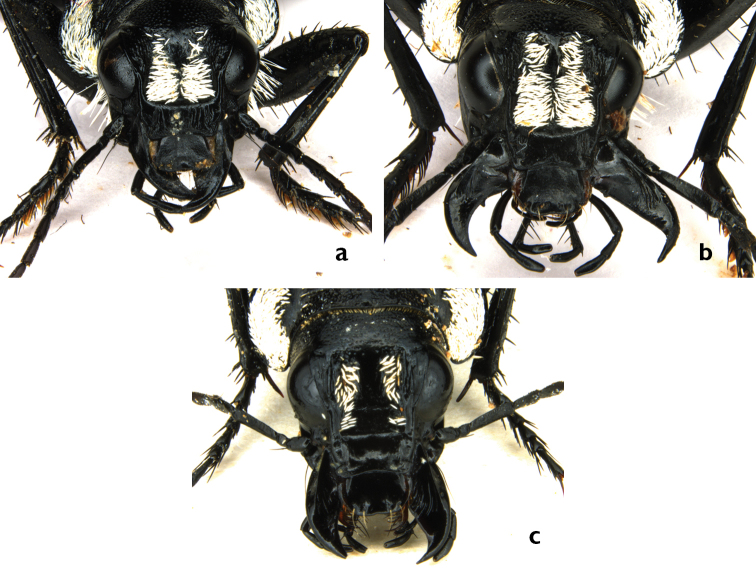
Frontal white stripes of white scales in both sexes: **a** Male: apical white frons stripes wider than exposed frons (*G. multiguttatus*) **b** Male: apical white frons stripes slender than exposed frons (*G. serrator*) **c** Female: sparse stripes of scales (*G. serrator*).

Frons in male with two stripes of white scales attached anteriorly to each other and diverging posteriorly from each other, leaving apical frons uncovered by white scales for a section wider or slenderer than one of the given stripes (Fig. 4a–c). This scale-free section is termed an ‘exposed frons’, and can also be raised to form a ridge.

**Figure 5.**
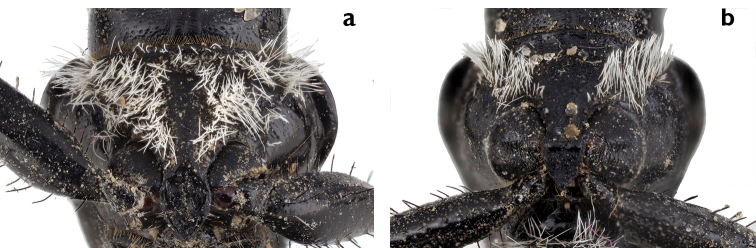
Ventral part of pronotum (*G. serrator*): **a** Male with dense white setae **b** Female with sparse white setae.

Pronotum strongly cordiform, wider anteriorly, narrower posteriorly; anterior margin sinuose, in the middle convex and shortly concave laterally to the protruding and rounded anterior angles; slight transverse anterior pronotal impression behind the middle of anterior margin; posterior margin concave; lateral margin sinuose. Median longitudinal impression slightly impressed medially, drawn to anterior and posterior margins, or sometimes absent. Lateral margin with white dense scales in the lateral bead, disc glabrous. Ventral side of the pronotum in males with dense white setae, in females with sparse white setae, less extended medially (Fig. 5a–b).

**Figure 6.**
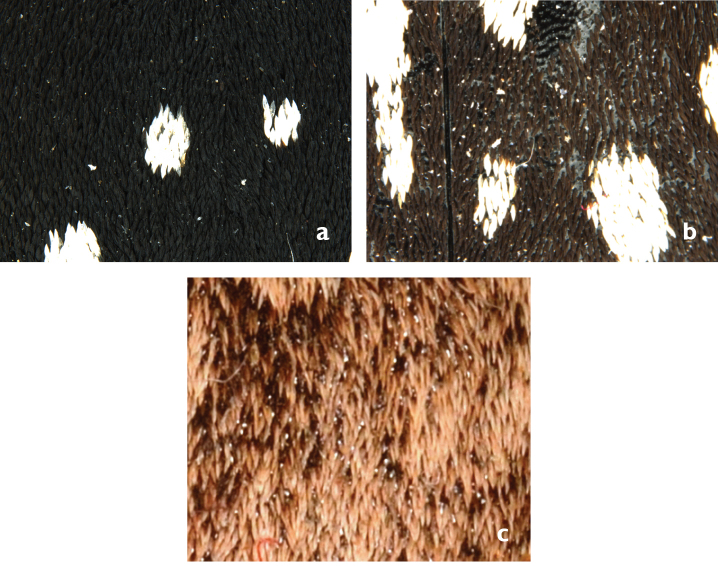
Elytral scale coloration: **a** Black dense scales (*G. serrator*) **b** Dark brown sparse scales (*G. reymondi*) **c** White slushy scales (*G. barthelemyi*).

**Figures 7.**
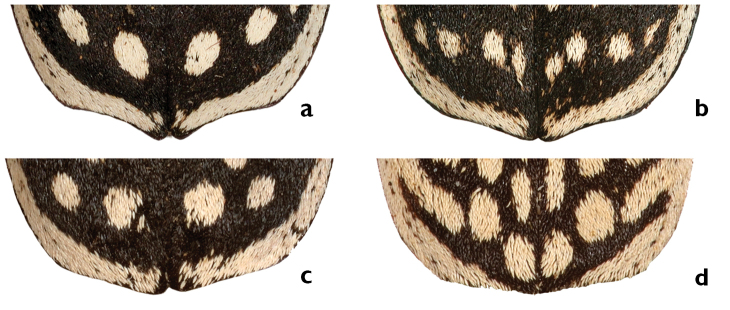
Apical section and apices of elytra: **a** Apical sinuation strongly developed, apex protruded, almost rectangular, only slightly rounded at most distant tip (*G. rotundatus*) **b** Apical sinuation developed, apex slightly protruded, strongly rounded (*G. luctuosus*) **c** Apical sinuation slightly developed to straight, apex not protruberant, broadly rounded, especially on the median side (*G. multiguttatus*) **d** Apical sinuation and apex almost indistinct (*G. minutus goryi*).

Elytra in most species oval, evenly rounded to drop-like shape, with isodiametric microsculpture and oval meshes, additionally covered by black or dark brown dense or sparse or white greyish scales (Fig. 6a–c). 2-6 marginal extensions of white dense scales originating from radial field posteriorly oriented and rounded toward suture, close to meet at apex. 10-40 white rounded or elongated spots on disc, in some species fused with lateral margin or with other spots to a complex pattern, umbilicate series of punctures extended with up to 15 thin bright setae (trichobotria), including the apical seta. Apical margin of each elytron sinuous to straight, posterolateral angle completely rounded, somewhat projecting; elytral apex slightly protruded, not protuberant or absent (Fig. 7a–d).

**Figure 8.**
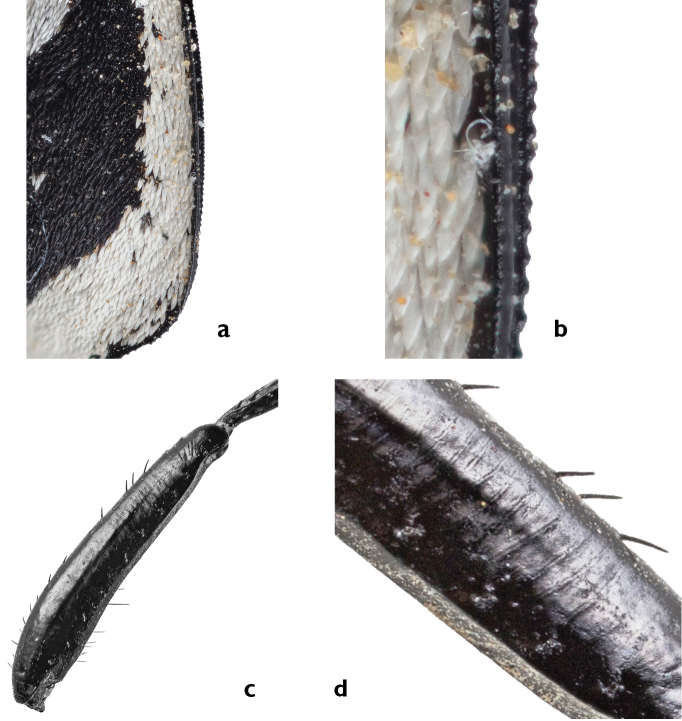
Stridulatory structure of *Graphipterus serrator*: **a** Serrated epipleural structure on the elytra edge **b** Magnification of a. **c** Carina on the upper side of the metafemur **d** Detail enlargement of **c**.

Within the genus *Graphipterus*, the stridulatory structure is a unique character for the *G. serrator* group, but does not occur in *G. minutus*. The structure consists of a serrated epipleural structure on the elytral lateral edge (Fig. 8a–b) and a carina situated on the upper side of the metafemur (Fig. 8c–d). The metatibia bears also a carina, but with some bristles. The latter ones are the reason why the metatibia does not function as part of the stridulatory structure. The carina on the metatibia occurs also in *G. minutus*, a species without the ability to stridulate, but it lacks the carina on the upper side of the metafemur. The chirping sound is created by rubbing the two hind leg femora on the elytra. The sound can be heard from a distance of several meters by the human ear.

**Figure 9.**
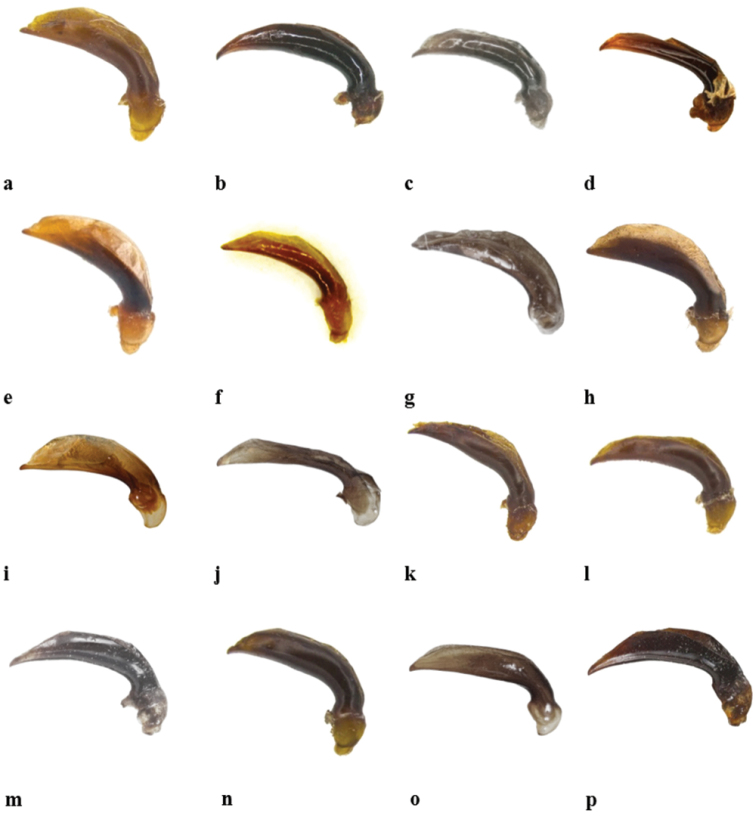
Median lobes of aedeagus: **a** *G. barthelemyi* **b** *G. heydeni* **c** *G. luctuosus* **d** *G. magnus* sp. n. **e** *G. mauretensis* sp. n. **f** *G. minutus minutus* **g** *G. minutus goryi* **h** *G. multiguttatus* **i** *G. peletieri* **j** *G. piniamitaii* sp. n. **k** *G. reymondi* **l** *G. rotundatus* **m** *G. serrator* **n** *G. sharonae* sp. n., **o** *G. stagonopsis* sp. n. **p** *G. valdanii*.

Shape of median lobe of aedeagus occurs in four types with different variations which can be used as diagnostic features (in contrast to Basilewsky’s 1977 claim). The four types are: tip sharp and ventrally bent (Fig. 9b, c, j, k, l, m, n, o, p); tip short and not bent (Fig. 9a, e, h, i), tip wide and flat (Fig. 9f, g), and tip thin and ventrally bent (Fig. 9d).

Ratios: HW/PW: 0.68–0.78, EYL/EL: 0.14–0.19. PL/PW: 0.54–0.72, BPWBPW/PW: 0.46–0.7, EL/EW: 1.08–1.29, EL/MTIL: 1.52–2.08, MTAL/MTIL: 0.72–1.28.

Taxonomic note: *Graphopterus* Agassiz, 1847 is a junior synonym of *Graphipterus* Latreille, 1802 (Basilewsky 1977; Lorenz 2005).

### Species accounts

#### Graphipterus barthelemyi

Taxon classificationAnimaliaColeopteraCarabidae

Dejean, 1830

Figs 3a , 6c , 9a , 17 , 20a–b

##### Types.

Holotype: ♂ (Blue label, black handwritten): <*Barthelemyi. Solier*/. in Barbaria. Tunis. D. Barthelemyi>. (White label with brown margin, brown letters, handwritten): <EX Musaeo/Chaudoir>. (Red label, black letters, type written): <TYPE>. Deposited in NHMB, Chaudoir collection [examined].

##### Diagnosis.

Medium-sized species with grayish or yellowish scales usually cover the elytra and sometimes also on the pronotum. Elytra pattern rarely visible with six lateral margin extensions and 18–24 isolated white circular to elongated spots occur on elytra.

##### Description.

BL male: 13.0–17.0 mm, average 15.8 ± 1.5 mm; BL female: 15.5–17.0 mm, average 16.3 ± 0.6 mm. Grayish with elytral white blurred spots and extensions.

*Head* slender: HW/PW: 0.72; EYL: 1.1-1.4 mm; EYL/EL: 0.15. Mentum without teeth (Fig. 3a). Frontal ridge reduced. In male, apical white frons stripes slenderer than exposed frons (cf. Fig. 4a).

*Pronotum* wide; PL/PW: 0.63; BPW/BPW/PW: 0.6; posteromedially concave and with white lateral margin, as wide as antennomere I long: white slushy scales cover disc sometimes.

*Elytra* wide, elytron margin almost continuously rounded from humeri to posterolateral angles; EL: 7.1–8.8 mm, average 8.2 mm; EW: 6.5–7.8 mm, average 7.3 mm; EL/EW: 1.1. Elytra longitudinally flat, usually with grayish scales, disc visible between scales (Fig. 6c); extensions and lateral margin blurred. Lateral margin nearly as wide as antennomere I long and with six extensions; extension I usually elongated; white posterior margin almost touches suture at apex. Disc with 18–24 rounded, usually elongated spots, anterior pair of spots elongate, as wide as extension I; posterior pair of spots rounded, located toward suture; round spots located posterior to third extensions laterally in imaginary lateral line as posterior spots. Apical sinuation slightly developed to straight, apex not protuberant, broadly rounded, especially medially (Fig. 7c). Suture inconspicuous.

*Legs* medium; MTIL: 4.5–6.5 mm, average 5.7 mm; El/MTIL: 1.7. Metatibial secondary spur brown. MTAL: 3.5–4.4 mm, average 4 mm; MTAL/MTIL: 0.8. Claws of hind legs brown at base.

Median lobe of *aedeagus* with short unbent tip (Fig. 9a).

##### Comparisons.

Distinguished from all other species of the *G. serrator* group by white lateral margins merged at the posterior margin of the pronotum. Median lobe of aedeagus with short, straight tip.

##### Habitat.

Unknown. The species was found exclusively in coastal dune habitats.

##### Co-occurring species.

*Graphipterus barthelemyi* lives in sympatry with *G. luctuosus* in Tunisia.

##### Distribution.

Restricted to north-east Tunisia (Fig. 17).

##### Conservation.

The restricted distribution range of the endemic species and the decline of the coastal sandy habitat as a result of increasing anthropogenic pressures (e.g., tourism activities, urbanization, etc.) threaten at least the long-term survival of the species.

##### Comments.

Both Basilewsky (1977) and Lorenz (2005) note in error that *G. barthelemyi* was described by Dejean (1831).

#### Graphipterus heydeni

Taxon classificationAnimaliaColeopteraCarabidae

Kraatz, 1890: 77, stat. rest.

Figs 3b , 9b , 18 , 21a, c

Graphipterus luctuosus Guérin-Méneville, 1859 (nec Dejean, 1825)

##### Types.

Lectotype: ♂ (Blue label with black margin, black handwritten): <*Heydeni Krtz./ luctuosus Guer./Tripolis.* Oued>. (White label, print black): <Coll. Kraatz>. (White label, black print): <Tripolis>. (White label, black print): *G. serrator*/heydeni Kr>. (Green label, black print): <Muncheberg/Col – 01309>. (White label, black print): serrator/ heydeni Kz./P. Basilewsky det., 1975>. Deposited in ZSM [examined].

Paralectotype: two specimens – ♂, ♀ (White label, black black handwritten): <Tripolis>. (White label, black handwritten): <Call. Kraatz>. (White label, black handwritten): <Muncheberg/Col – 01310/01311>. Deposited in ZSM [examined]. Lectotypes and paralectotypes herewith designated.

##### Diagnosis.

Large species with 18–26 isolated white round spots on elytra, anterior and posterior discal spots larger than other spots; four marginal extensions, anterior extension triangular; median lobe of aedeagus with ventrally bent apex.

##### Comparisons.

*Graphipterus heydeni* resembles *G. valdanii* from which it differs mainly by the following characters: *G. heydeni*: mentum with two teeth, margin between them clearly concave; EL/EW rounded (1.24); 18-26 spots on elytra; claws of hind legs dark; metatibial secondary spur brown. In *Graphipterus valdanii*, mentum with two teeth, margin between them slightly convex in middle; EL/EW elongated (1.31); 18-26 spots on elytra; claws of hind legs brown; metatibial secondary spur dark. *Graphipterus heydeni* also resembles *G. magnus* sp. n. from which it differs mainly by the following characters: *G. heydeni*: elytra shape oval; four elytral marginal extensions; anterior and posterior elytral spots larger than all other spots; median lobe of aedeagus with stout with ventrally bent tip. *G. magnus* sp. n.: elytra shape rounded; six elytral marginal extensions; all elytral spots with similar size; median lobe of aedeagus elongated with ventrally bent tip.

##### Description.

BL male: 17.1–20.9 mm, average 18.9 ± 1.6 mm; BL female: 18–20 mm, average 19.4 ± 1.4 mm.

*Head* slender; HW/PW: 0.71; EYL: 1.5–1.9 mm; EYL/EL: 0.16. Mentum with two teeth and concavity between them (Fig. 3b). Frontal ridge absent. In male, apical white frons stripes slenderer than exposed frons (Fig. 4a).

*Pronotum* slender; PL/PW: 0.65; BPW/PW: 0.7; posteromedially concave and without white margin; white lateral margin as wide as antennomere I long.

*Elytra* oval, humeri rounded; EL: 10.0–12.1 mm, average 10.9 mm; EW: 8.4–9.9 mm, average 9.16 mm; EL/EW: 1.2. Lateral cross section convex. Black scales dense, disc not visible between them (Fig. 6a). White lateral margin almost as wide as antennomere I long and with four extensions; extension I triangular with rounded angels, slightly wider at margin of elytra, slightly elongated, wider and shorter than extension II; the latter one elongated at third quarter of elytra, imaginary line connecting the media ends of the extensions I and II parallel to the suture; white posterior margin forms a gap at suture, wider than lateral margin. Disc usually with 18–26 rounded spots; anterior pair of spots rounded, as wide as extension I, usually smaller than posterior spots, larger than spots on mid disc; mid disc spots usually asymmetrically smeared. Posterior pair of spots rounded, one or two small spots located laterally to posterior spots. Apical sinuation strongly developed, apex protruded, almost rectangular, only slightly rounded at most distant tip (Fig. 7a). Suture inconspicuous.

*Legs* long; MTIL: 5.3–7.0 mm, average 6.5 mm; El/MTIL: 1.7. Metatibial secondary spur brown. MTAL: 4.4–5.2 mm, average 4.8 mm; MTAL/MTIL: 0.7. Claws of hind legs black at base.

Median lobe of *aedeagus* with apex bent ventrally (Fig. 9b).

##### Habitat.

Unknown.

##### Co-occurring species.

*Graphipterus heydeni* lives in sympatry with *G. luctuosus* around Tripoli, Libya, and might live in sympatry with *G. rotundatus* in this region. It also lives in sympatry with *G. piniamitaii* sp. n. in Nefzaoua region in Tunisia.

##### Distribution.

Western Lybia (Tripolitania) and western Tunisia (Nefzaoua) (Fig. 18).

##### Conservation.

The restricted distribution range of the endemic species and the decline of the coastal sandy habitat as a result of increasing anthropogenic pressures (e.g., tourism, urbanization etc.) threaten at least the long-term survival of the species.

##### Comments.

This taxon was first described by Guérin-Méneville (erroneously as *luctuosus* Dej.). As Kraatz already noted, Guérin-Méneville's and Dejean's specimens do not belong to the same taxon, and Kraatz substituted the name *heydeni* Kraatz, 1890 as a new replacement name (nomen novum) for the already available name *luctuosus* Guérin-Méneville. However, Kraatz never fixed the holotype (Jäger, pers. comm.), following the requirement of Article 72.2 (ICZN 1999). The type series of *heydeni* comprises three individuals from Tripoli (Kraatz 1890: 77) and not seven (holotype and six paratypes) as indicated by Basilewsky (1977: 451). The beetles were collected by Quedenfeldt, as this circumstance was indicated by Kraatz in the original description. These individuals have been transferred to the DEI (Kraatz was the director of this institution) and the syntypes are still preserved there. A lectotype is designated, labeled with a handwritten card indicating the taxon’s name, the name of the location, Tripoli, and the initial letters of the collector (Fig. 21c). The above description is based primarily on the three syntypes. The misinterpretation of the type material by Kraatz led Basilewsky to an incorrect interpretation of *heydeni* Kraatz. Consequently the distribution map given by Basilewsky (1977: page 450) is also incorrect.

#### Graphipterus luctuosus

Taxon classificationAnimaliaColeopteraCarabidae

Dejean, 1825: 335

Figs 7b , 9c , 16 , 21b

Graphipterus reichei Guérin-Méneville, 1859: 534 (Tripoli)
Graphipterus intermedius Guérin-Méneville, 1859: 534 (Tripoli)

##### Types.

Holotype: ♂ (Green label, black handwritten): <*Luctuosus. mihi*/ *h. in* Barbaria. Tripoli>. (White label, black typewritten): <P. Bedel/Visit 1905>. (White label with brown margin, brown typewritten): <EX Musaeo/Chaudoir>. (Red label, black typewritten): <TYPE>. Deposited in NHMB, Chaudoir collection [examined]. Neotype: ♂ (White label, black handwritten): <Tripolis>. (White label, black handwritten): <Coll: Kraatz>. (Green label, black handwritten): <DEI Muncheberg/ Call-01342>. (Red label, black typewritten): <Neotypus/*Graphipterus reichei*/ Guérin-Méneville, 1859/ des. I. Renan, 2018. Neotype: ♂ (White label, brown handwritten): <intermedius/♂ reichei>. (White label, brwon handwritten): <Tripolis>. (White label, black handwritten): <Call: Kraatz>. ♂ (Green label, black handwritten): <DEI Muncheberg/Call-01343>. (Red label, black typewritten): <Neotypus/*Graphipterus intermedius*/Guérin-Méneville, 1859/des. I. Renan, 2018>.

##### Diagnosis.

Medium-sized species with 22–30 isolated white, usually elongated elytral spots, six very short marginal extensions, and a series of 8–12 elongated spots along suture form a broken line. Median lobe of aedeagus with apex slightly bent ventrally.

##### Comparisons.

*Graphipterus luctuosus* resembles *G. peletieri* from which it differs mainly by the following characters: *G. luctuosus*: apical white frons stripes, wider than exposed frons; six elytral extensions; 8–12 elongated spots along suture of elytra; elytral suture conspicuous; white posterior margin almost attached; median lobe of aedeagus with bent tip. *G. peletieri* apical white frons stripes slenderer than exposed frons; four elytral extensions; elytral spots scattered; elytral suture not conspicuous; white posterior margin forming gap; median lobe of aedeagus with unbent tip. *Graphipterus luctuosus* resembles also *G. rotundatus* from which it differs mainly by the following characters: *G. luctuosus*: 8–12 elongated spots along suture of elytra; scales of elytral disc brown, disc visible between; metatibial secondary spur brown. *G. rotundatus*: elytral spots scattered; scales of elytral disc black, disc not visible between them; metatibial secondary spur dark, not darker than the elytral scales.

##### Description.

BL male: 15.0–17.5 mm, average 15.8 ± 1.5 mm; BL female: 15–18 mm, average 16.3 ± 0.6 mm.

*Head* medium; HW/PW: 0.74; EYL: 1–1.6 mm; EYL/EL: 0.15. Mentum with two or three teeth. Frontal ridge absent. In male, apical white frons stripes wider than exposed frons, (cf. Fig. 4b).

*Pronotum* cordiform; PL/PW: 0.64; BPWBPW/PW: 0.61; posteromedially concave and without white margin; white lateral margin as wide as antennomere I long.

*Elytra* oval, humeri rounded; EL: 7.3–9.9 mm, average 8.9 mm; EW: 5.8–8.4, average 7.7 mm; EL/EW: 1.16. Lateral cross section quite flat suture conspicuous. Scales brown, disc visible between them (Fig. 6b). White lateral margin narrow, as wide as half antennomere I long and with six extensions, rarely four; extension I usually elongated, sometimes constricted at the base to the lateral margin; extension II elongated, constricted or absent; extension III elongated; white posterior margin almost as wide as the lateral margin, gap at suture smaller than lateral margin or even absent. Disc usually with 22–30 mostly elongate small spots; anterior pair of spots slightly elongate, wide as extension I, lateral spots rounded, adjacent or sometimes fused to extension II, posterior pair of spots rounded, slightly larger than others, located toward suture, round spots slightly smaller than all others, located posteriori to third extensions and laterally to posterior spots; a series of 8–12 elongated spots along the suture. Apical sinuation developed, apex slightly protruded, strongly rounded (Fig. 7b).

*Legs* medium; MTIL: 4.5–5.9 mm, average 5.4 mm; El/MTIL: 1.7. Metatibial secondary spur brown. MTAL: 3.4–4.7 mm, average 4.4 mm; MTAL/MTIL: 0.8. Claws of hind legs brown at base.

Median lobe of *aedeagus* with ventrally bent tip (Fig. 9c).

##### Habitat.

Unknown.

##### Co-occurring species.

*Graphipterus luctuosus* lives in sympatry with seven other species: *G. peletieri* in north-west Algeria, *G. heydeni* in Tripoli region, *G. valdanii* in north Algeria, *G. rotundatus* in Tunisia and Algeria, *G. stagonopsis* in the Ghardaia region, Algeria, *G. piniamitaii* sp. n. in Tunisia, and *G. barthelemyi* in north-east Tunisia.

##### Distribution.

*Graphipterus luctuosus* presents the widest distribution range of the group: from Laghouat, more than 300 km inland Algeria to the arid and semi-arid regions of north-east Algeria, over most of the Tunisian coast and east up to Sirte on the Libyan coast (Fig. 16).

##### Conservation.

The species does not seem to be endangered as it apparently lives in numerous habitats. Consequently, it might not be so strongly affected by human activities.

##### Comments.

*Graphipterus reichei* and *G. intermedius* have been described by Guérin-Méneville (1859) as variants of *G. multiguttatus*. Unfortunately, the type material of both Guérin-Méneville's taxa has have been lost. Neotypes for both taxa are designated. Based on the original description and the type locality, Tripoli, the only other known species from the type locality is *G. heydeni*, which is clearly different in elytral pattern and body length. Basilewsky (1977), Huber and Marggi (2017) and Lorenz (2005) ranked both taxa as synonyms of *G. luctuosus*.

#### Graphipterus magnus

Taxon classificationAnimaliaColeopteraCarabidae

Renan & Assmann sp. n.

http://zoobank.org/EFC7478C-E761-43C8-901F-3FEE1B8A7998

Figs 9d , 19 , 22a

##### Types.

Holotype: ♂ (White label, black handwritten): <23.II 1942/Buq Buq/P.J. Gent/Egypt> (White label, black typewritten and black handwritten): <Brit. Mus./1952-180> (White label, black typewritten): <BMNH {E}/UIN989817>. (ae) Deposited in BMNH [examined].

Paratypes: (2 ♂), Egypt, Buq Buq: 14.11.1942, P.J. Gent, {E}/UIN989815 (♂); Egypt, E. of Buq Buq, 14.11.1942, P.J. Gent, {E}/UIN989815, Brit. Mus.952-180 (♂) (BMNH).

##### Diagnosis.

Large species with 20–24 white rounded and elongated elytra spots; six white marginal extensions, extension I elongated. Elytra wide, lateral margin strongly and continuous rounded. Aedeagus elongated, thin and with apex slightly bent ventrally (Fig. 9d).

##### Comparisons.

*Graphipterus magnus* sp. n. resembles *G. heydeni* from which it differs mainly by elytra shape and pattern, and aedeagus shape (see comparisons in *G. heydeni*).

##### Description.

BL male: 18.3–20.1 mm, BL female: unknown. Average 19.4 ± mm.

*Head* slender; HW/PW: 0.72; EYL: 1.7 mm; EYL/EL: 0.17. Frontal ridge well developed. In male, apical white frons stripes slenderer than exposed frons (cf. Fig. 4a). Pronotum cordiform; PL/PW: 0.62; BPW/BPW/PW: 0.68; posteromedially concave and without white margin; white lateral margin as wide as antennomere I long.

*Elytra* wide, rounded, rounded-like, humeri strongly narrowed; EL: 9–10.7 mm, average 9.7 mm; EW: 8.5–9.0 mm, average 8.7 mm; EL/EW: 1.1. Lateral cross section quite flat. Scales black, disc not visible between them (cf. Fig. 6a). White lateral margin nearly as wide as antennomere I long and with six extensions; extensions I slightly elongated, wider close to the margin; extensions II and III in front of middle. White posterior margin as wide as lateral margin or wider, sutural gap slenderer than lateral margin. Disc usually with 20 (rarely up to24) spots; anterior pair of spots rounded, wider than extension I, 6–8 spots adjacent elongated and parallel to suture, posterior pair of spots rounded, additional 1–3 small spots frequently present laterally to posterior ones. Apical sinuation strongly developed, apex protruded, almost rectangular, only slightly rounded at most distant tip (Fig. 7a). Suture conspicuous.

*Legs* long; MTIL: 6.2–6.8, average 6.5 mm; El/MTIL: 1.53. Metatibial secondary spur brown. MTAL: 5.2mm; MTAL/MTIL: 0.8. Claws of hind legs brown at base.

Median lobe of *aedeagus* long and thin with apex hardly bent ventrally (Fig. 9d).

##### Etymology.

The species name is derived from Latin (*magnus*) and refers to the large body size.

##### Habitat.

Unknown.

##### Co-occurring species.

No co-occurring species.

##### Conservation.

Unknown.

##### Distribution.

The only known records are from Buq Buq in north-east Egypt (Fig. 19).

#### Graphipterus mauretensis

Taxon classificationAnimaliaColeopteraCarabidae

Renan & Assmann sp. n.

http://zoobank.org/F3446E7A-90BB-47EA-9D08-109144005B66

Figs 9e , 16 , 22b

##### Types.

Holotype: ♂ (White label with pencil handwritten) < luctuosus/(uc)>. (White label with black typewritten and handwritten): <OFFICE NATIONAL ANTIACRIDIEN/Azefal Mauritania/13 Fevirer 1950/J. Leroux>. (red label): <Holotype> (ae). Deposited in Colas collection, NHMB.

Paratypes: (3 ♂, 1♀), Azefal Mauritania: 13 Fevrier 1950, J. Leroux (♂) (NHMB, Colas collection): Mauritanie [Mauritania]: Chingvetti, 3.1951, L. Dekeyser and A. Villiers (2♂ (1-ae), ♀) (MRAC).

##### Diagnosis.

Medium-sized species with (18–) 22 white, mostly elongated spots on elytra, anterior and posterior spots larger than other spots; six marginal extensions, extension I usually triangular. Median lobe of aedeagus with short apex unbent ventrally (somewhat similar to that of *G. barthelemyi*).

##### Comparisons.

*Graphipterus mauretensis* sp. n. resembles *G. piniamitaii* sp. n., from which it differs mainly by the following characters: *G. mauretensis* sp. n.: (18–) 22 spots on elytra; anterior and posterior elytral spots larger than all other spots; apical sinuation and apex developed and slightly protruded; median lobe of aedeagus with short bent tip. *G. piniamitaii* sp. n.: 24 spots on elytra; only posterior elytral spots larger than all other spots; apical sinuation and apex strongly developed and protruded; median lobe of aedeagus with ventrally bent tip.

##### Description.

BL male: 15.1–17.5 mm, average 16.6 ± 1.1 mm. Females were not available.

*Head* medium; HW/PW: 0.76; EYL: 1.3–1.5 mm; EYL/EL: 0.16. Mentum with two teeth (cf. Fig. 3b). Frontal ridge reduced. In male, apical white frons stripes wider than exposed frons (cf. Fig. 4b).

*Pronotum* cordiform; PL/PW: 0.66; BPW/PW: 0.63; posteriomedially concave and without white margin; white lateral margin as wide as antennomeres I+II long.

*Elytra* relatively elongated oval humeri slightly narrowed; EL: 8.6–9.7 mm, average 9.2 mm; EW: 6.8–8.0 mm, average 7.4 mm; EL/EW: 1.2. Lateral cross section quite flat. Dense black scales, disc not visible between them (Fig. 6a). White lateral margin as wide as half of antennomere I long and with six extensions; extension I triangular; extension II shorter than extension III. White posterior margin as wide as lateral margin, sutural gap slenderer than lateral margin. Disc usually with 18–22 rounded to elongate spots; anterior spot elongated, as wide as extension I, anterior and posterior spots larger than all other ones, posterior one rounded. Apical sinuation developed, apex slightly protruded, strongly rounded (Fig. 7b). Suture conspicuous.

*Legs* long; MTIL: 5.3–5.9 mm, average 5.7 mm; El/MTIL: 1.61. Metatibial secondary spur brown at base, MTAL: 3.8–4.5 mm, average 4.2 mm; MTAL/MTIL: 0.74. Claws of hind legs brown at base.

Median lobe of *aedeagus* with short apex, unbent ventrally (Fig. 9e).

##### Etymology.

The species name is derived from ancient Latin (Mauretania, -ensis).

##### Habitat.

Unknown.

##### Co-occurring species.

No co-occurring species.

##### Distribution.

As we found in all collections only nine specimens of *G. mauretensis* sp. n., our knowledge of its distribution range is limited. Known from central coast of Mauritania to more than 400 km inland to Glebat el M’Boza Adrar (Fig. 16).

##### Conservation.

Unknown.

#### Graphipterus minutus minutus

Taxon classificationAnimaliaColeopteraCarabidae

Dejean, 1822: 96

Figs 3c , 9f , 11 , 19 , 23a

##### Types.

Lectotype: ♀ (blue label, black handwritten): <minutus. m/ h. in Egypt>. (blue label, black handwritten): <Olivier>. (White label with brown margin, brown typewritten): <EX Musaeo/Chaudoir>. (Red label, black typewritten): <TYPE>. Deposited in BMNH, Chaudoir collection [examined].

Paralectotypes: ♀ (blue label, black handwritten): <*Graphipterus* {*minutus*. Ol./minutus. Dej./Egypt. C. Olivier>. (Green circular label with black margin, black typewritten): <COLLECTION/OLIVIER/TYPE>. Deposited in BMNH, Chaudoir collection [examined]. ♀ (Green circular label with black margin, black typewritten): <COLLECTION/OLIVIER/TYPE>. Deposited in BMNH, Olivier collection [examined].

Additionally, two syntypes are deposited in Chaudoir’s collection, NHMB [examined].

##### Diagnosis.

The two subspecies of *G. minutus* are distinguished from all other species of the *G. serrator* group by smaller size, lack of the stridulatory structure, unique pronotum shape (*G. serrator* group excluding *G. minutus*: BPW/PW: 0.6-0.7, *G. minutus*: BPW/PW: 0.46) and flat tip of median lobe.

**Figure 10.**
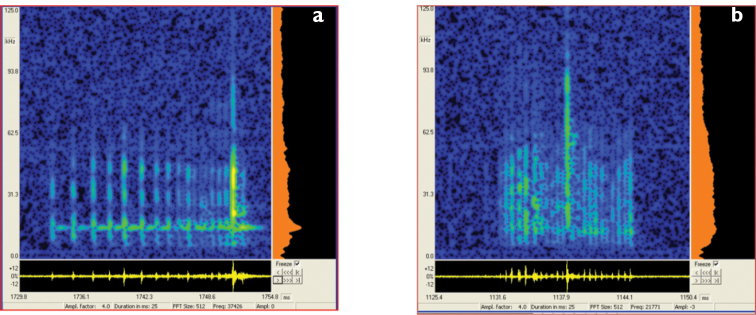
Spectrograms of two *Graphipterus* species: *G. serrator* and *G. multiguttatus*.

##### Comparisons.

*Graphipterus minutus minutus* differs from *G. minutus goryi* mainly by the following characters: *G. minutus minutus*: frontal ridge not developed; 36–40 spots on elytra; two elytra marginal extensions; rounded and separated spots along median suture. *G. minutus goryi*: 28–30 spots on elytra; six elytra marginal extensions; elongated and fused spots along median suture.

##### Description.

BL male: 10.3–13.5 mm, average 12 ± 1.2 mm; BL female: 10.5–15.2 mm, average 13.1 ± 1.9 mm;

*Head* wide; HW/PW: 0.77; EYL: 1–1.8 mm; EYL/EL: 0.15. Frontal ridge slightly developed. Male with two short parallel frontal stripes of white scales usually diverging apically, became wispy, not reach the level of supraorbital setiferous pores. Mentum usually with two pronounced teeth (Fig. 3e). Pronotum strongly cordiform PL/PW: 0.54; BPW/PW: 0.46; posteromedially flat and without white margin; white lateral margin as twice as antennomere II long.

*Elytra* almost rounded, humeri stringly rounded, lateral margin continuously rounded; EL: 5.3–7.5 mm, average 6.6; EW mm: 4.8–7.6 mm, average 6.1 mm; EL/EW: 1.16. Suture inconspicuous. Scales black, disc not visible between them (cf. Fig. 6a). Lateral cross section convex. Apical sinuation almost lacking, apex almost absent, not rounded (Fig. 7d). White lateral margin usually nearly as wide as antennomere I long and usually with two extensions; extension I elongated from humeri posteriorly; extension II usually absent, sometimes indistinct wider section of lateral margin at its middle. White posterior margin becomes narrower toward the tip, usually disappearing in front of it; gap at suture wider than lateral margin. Disc with 36–40 mostly rounded spots; usually 12, sometimes ten or 14 rounded to elongated, not fused spots located parallel to suture; anterior spot as wide as extension I. Stridulatory structure absent.

*Legs* short; MTIL: 2.54–4.0 mm, average 3.3 mm; El/MTIL: 1.9 mm. Metatibial secondary spur brown, MTAL length: 2.5–3.3 mm, average 2.9 mm; MTAL/MTIL: 0.85. Claws of hind legs brown at base.

Median lobe of *aedeagus* with wide and flat tip (Fig. 9f).

##### Habitat.

**Figure 11.**
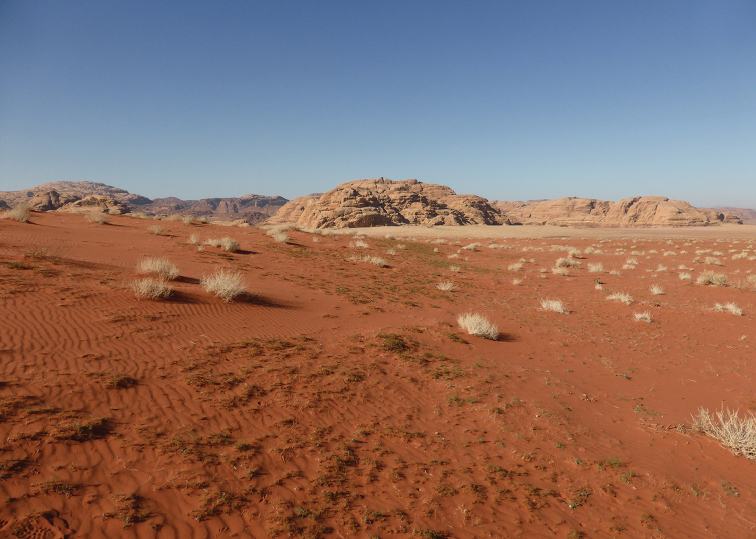
Habitat of *Graphipterus minutus minutus*: Shallow sand dunes in Wadi Ram, Jordan.

Sparse populations in arid habitats with hallow sand dunes, and scant shrubs landscape (Fig. 11).

##### Co-occurring species.

No co-occurring species.

##### Distribution.

Syria, east and south Jordan, north Saudi Arabia, Iraq, and Iran (Fig. 19).

##### Conservation.

The species does not seem to be endangered as it has a wide distribution range that is not strongly affected by human activities.

##### Comments.

The type location of *G. m. minutus*, Egypt, is probably a labeling mistake. Only four specimens of this species were found with labels from Egypt; the three syntypes from Olivier’s collection and one specimen deposited in BMNH, collected by Bowring. Even though Olivier had a large amount of material from Egypt and Bowring collected in Egypt, we are convinced that *G. minutus* does not occur in Egypt: all the known populations from collections and field observations are from Asia and not from Africa. Furthermore, no specimen has been ever collected in Israel, despite intensive collecting in the potential habitats. Basilewsky (1977) noted that although several researchers had contended that *G. minutus* does exist in Egypt, they were wrong, but he does not refer to the problem of types.

By applying other species concepts (e.g., Evolutionary or Phylogenetic Species Concept, Claridge et al. 1997) or by using another approach to delineate species, the two taxa *minutus* and *goryi* might be ranked as two species. However, our numerical approach to delineate species results in a value for both *minutus* and *goryi* that is clearly below the threshold of the least differentiated sympatrically occurring species of the *Graphipterus serrator* group. Therefore these two taxa must be ranked as one species. Nonetheless both taxa differ clearly from each other and are well established in the literature as subspecies (Basilewsky 1977; Lorenz 2005; Huber and Marggi 2017). Therefore we prefer a conservative taxonomic approach which avoids taxonomic inflation (cf. Zachos et al. 2013, Assmann et al. 2008) and preserve the rank of subspecies for both taxa.

#### Graphipterus minutus goryi

Taxon classificationAnimaliaColeopteraCarabidae

Chaudoir, 1848: 127

Figs 7d , 9g , 19 , 23b

##### Types.

Holotype: ♂ (White label with brown margin, brown typewritten): <EX Musaeo/Chaudoir>. (Red label, black typewritten): <TYPE>. Deposited in BMNH, Chaudoir collection [examined].

##### Diagnosis.

Small-sized taxon with 28–30 mostly elongated white spots, usually with several spots fused with lateral margin, and with series of usually ten elongated spots, regularly at least several are fused to each other along median suture. Two marginal extensions elongated from humeri posteriorly. Median lobe of aedeagus with wide and flat tip.

##### Comparisons.

*Graphipterus minutus goryi* resembles *G. minutus minutus*, for further details see *Graphipterus minutus minutus*.

##### Description.

BL male: 11.2–11.8 mm, average 11.5 ± .02 mm; BL female: 11.4–13.6 mm, average 12.2 ± 0.9 mm.

*Head* wide; HW/PW: 0.78; EYL: 1.1–1.3 mm; EYL/EL: 0.19. Frontal ridge absent. Male with two short parallel frontal stripes of decumbent white scales usually diverging apically became wispy, not reaching the level of supraorbital setae. Mentum with two pronounced teeth (cf. Fig. 3e).

*Pronotum* strongly cordiform; PL/PW: 0.57; BPW/PW: 0.5; posteriomedially flat and without white margin; white lateral margin as twice as antennomere II long.

*Elytra* almost rounded, humeri strongly rounded, lateral margin continuously rounded; EL: 5.9–7.3 mm, average 6.2 mm; EW: 5.2–6.8 mm, average 5.7 mm; EL/EW: 1.1. Lateral cross section convex. Scales black, disc not visible between them (cf. Fig. 6a). White lateral margin nearly as wide as antennomere I long and with six, rarely fewer, elongated extensions; extensions I elongated from humeri posteriorly; extensions II and III in front and behind the middle of lateral margin, usually much longer than lateral margin wide. White posterior margin becomes narrower toward the tip, usually disappearing in front of it; gap at suture as wide as lateral margin. Disc with 28–30 mostly elongated spots, several spots fused with lateral margin resulting in extensions II and III, a series of 10, (rarely 12–14), elongated spots fused to each other parallel to suture, anterior spot as wide as extension I. stridulatory structure absent. Apical sinuation almost lacking, apex almost absent, not rounded (Fig. 7d). Suture inconspicuous.

*Legs* short; MTIL: 2.5–3.3 mm, average 3.0 mm; El/MTIL: 1.9. Metatibial secondary spur brown. MTAL: 2.1–3.8 mm, average 2.9 mm; MTAL/MTIL: 0.87. Claws of hind legs brown at base.

Median lobe of *aedeagus* with wide and flat tip (Fig. 9g).

##### Habitat.

Unknown.

##### Co-occurring species.

No co-occurring species.

##### Distribution.

Saudi Arabia and Iraq (Fig. 19). There are old records from Iran (Perse), but without indication of exact locality.

##### Conservation.

The species does not seem to be endangered as it has a wide distribution range in desert regions that are not strongly affected by human activities.

#### Graphipterus multiguttatus

Taxon classificationAnimaliaColeopteraCarabidae

(Olivier, 1790) 335, stat. rest.

**Figure 12.**
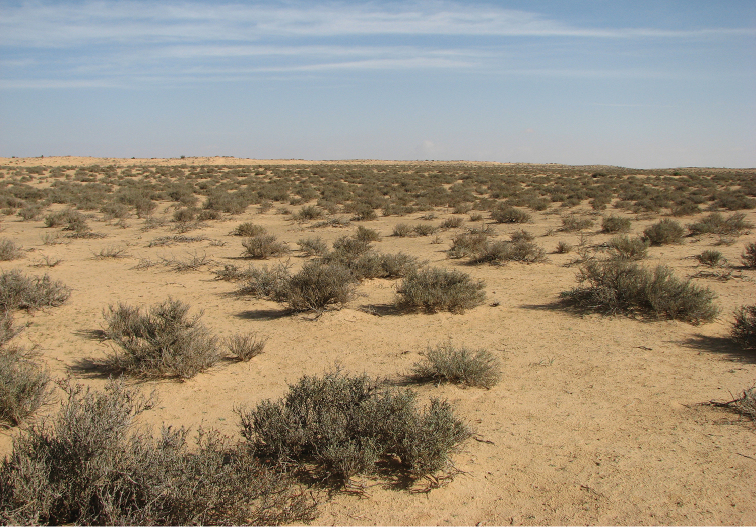
Habitat of *Graphipterus multiguttatus*: Sand rich with loess soil, relatively rich in annual plants. Western Negev sands, Israel.

Figs 4a , 7c , 9h , 10b , 12 , 19 , 24a

Graphipterus kindermanni Chaudoir, 1871: 299, syn. n. Alexandrie (= Alecsandria)

##### Types.

Lectotype: ♂ (blue label, black handwritten): <Graphipterus/multiguttatus. Ol./Egypt. *G. Olivieir*> (Green circular label with black margin, black typewritten): <COLLECTION/OLIVIER/TYPE>. Deposited in NHMB, Olivier collection [examined]. Syntypes: NHMB (Olivier collection): Egypte Olivier, multiguttatus, (uc), TYPE (♂); (Olivier collection) Collection Olivier, TYPE (♂); (General collection) Egypte Olivier, multiguttatus, Egypt, Oliv., Bedel et (uc), p. 339, 1909, vid. (♀).

##### Diagnosis.

Small species with 16–20 white, mostly elongated spots on elytra, only posterior discal spots rounded; 4–6 marginal extensions, extension I oriented slightly posteriorly. Median lobe of aedeagus with ventrally short, unbent tip.

##### Comparisons.

*Graphipterus multiguttatus* resembles *G. rotundatus* from which it differs mainly by the following characters: *G. multiguttatus*: average body length of 13.2 mm; El/MTIL, 1.6; all elytral spots with similar size; MTAL/MTIL, 0.84; median lobe of aedeagus with ventrally short unbent tip. *G. rotundatus*: average body length of 17.4 mm; El/MTIL, 2.08; posterior elytral spots larger than all other spots; MTAL/MTIL, 1.28; median lobe of aedeagus with longer (than *G. multiguttatus*) slightly bent tip. *Graphipterus multiguttatus* resembles also *G. sharonae* sp. n., from which it differs mainly by body length, elytral pattern, and shape of median lobe of aedeagus (see full comparisons under *G. sharonae* sp. n.).

##### Description.

BL male: 10.0–15.0 mm, average 13.0 ± 1.3 mm; BL female: 11.5–16.0 mm, average 14.0 ± 1.2 mm.

*Head* wide; HW/PW: 0.76; EYL: 1.0–1.6 mm; EYL/EL: 0.17. Frontal ridge slightly developed. In male, apical white frons stripes wider than exposed frons (Fig. 4a); stripes elongate, reaching the level of supraorbital setae (populations east of the Dead Sea-Rift Valley), or being shorter (populations west of the Dead Sea-Rift Valley). Mentum with 2–3 teeth.

*Pronotum* cordiform; PL/PW: 0.66; BPW/PW: 0.64; posteromedially concave and without white margin; white lateral margin as wide antonomer 1 long.

*Elytra* oval, humeri rounded; EL: 4.5–9.1 mm, average 7.7 mm; EW: 4.1–8.0 mm, average 6.4 mm; EL/EW: 1.2. Lateral cross section quite flat. Elytra with Dense black scales, disc not visible between them (cf. Fig. 6a). White lateral margin nearly as wide as half of antennomere I long and with 6, sometimes four extensions; extension I medium long, shorter than anterior spot, but longer than extension II and shorter than extension III, which is wider than lateral margin; extension II sometimes constricted, rarely absent or fused with lateral disc spot. White posterior margin as wide as lateral margin, gap at suture smaller than lateral margin. Disc usually with 16 sometimes 18 rounded to elongate spots; anterior spot slightly elongate, longer than extension I; lateral spots rounded, adjacent, or sometimes fused to extension II, six spots forming an arch pattern anteriorly and laterally to posterior rounded larger spots. Apical sinuation slightly developed to straight, apex not protuberant, broadly rounded, especially on the medial side (Fig. 7c). Suture conspicuous.

*Legs* long; MTIL: 3.7–5.5 mm, average 4.7 mm; El/MTIL: 1.6. Metatibial secondary spur brown at base, MTAL: 3–4.5 mm, average 3.7 mm; MTAL/MTIL: 0.8. Claws of hind legs black at base.

Median lobe of *aedeagus* with short, unbent tip (Fig. 9h).

##### Habitat.

In the western Negev (Israel), the species shows a significant habitat preference for stabilized interdunes and for the semi-stabilized slopes. In this region it is completely absent from the crest of shifting sand dunes. On the dunes it prefers the lower part of the north-facing slope, which is the part of the dune being most humid and most vegetated by annual plants (Fig. 12). Large populations inhabit the loamy and more humid region in the northern Negev. In spring, after an extremely dry winter, specimens might also be found on the margins of irrigated agriculture fields.

##### Co-occurring species.

*Graphipterus multiguttatus* lives in sympatry with *G. serrator* in Egypt and Israel.

##### Distribution.

Egypt, Israel, and Jordan (Fig. 19). The only Jordanian population of which we are aware lives between Aqaba to Ma’an, and inhabits a flat sand dune area without or only slightly developed crust. In the same habitat Anthia (Thermophilum) duodecimguttata (Bonelli, 1813) and *Amara maindromi* Bedel, 1907 occur.

##### Conservation.

The species does not seem to be endangered as it has a wide distribution range and it prefers habitats that are not strongly affected by human activities. However, in Israel, in the Dead Sea region and the Arava Valley, *G. multiguttatus* has been collected in the past, but no longer exists there. The latest records from these regions are Ein Gedi, 1976; Ein Husub, 1956 (leg. unknown, both specimens preserved in KCE); Sedom road, 1953; Ein-Radian, 1958 (leg. Ch. Lewinsohn, both specimens preserved in SMNHTAU). Habitats for *G. multiguttatus* on the Israeli side of the Arava valley may have disappeared. Anthia (Thermophilum) duodecimguttata (Bonelli, 1813), one of the co-occurring ground beetle species of the Jordanian population of *G. multiguttatus*, was last found in 2003 in Israel (coll. U. Shanas, V. Chikatunov, SMNHTAU; pers. obs.).

##### Comments.

Specimens from Jordan and the central Negev in Israel are usually larger than those from the western Negev. Specimens from the HaBesor National Park are smaller than those from the western Negev. The latter populations of *G. multiguttatus* which co-occur with *G. serrator* populations (Renan et al. 2011) have individuals with intermediate body lengths.

*Graphipterus kindermanni* has to be ranked as a junior synonym of *G. multiguttatus*. We checked for comparison the types of Basilewsky in MRAC (but did not find the type in NHMB that Basilewsky noted he had checked there) and did not find any morphological differences, with the exception of white setae on the elytral base. Both Basilewsky (1977) and Lorenz (2005) contended that *G. kindermanni* is a synonym of *G. luctuosus*.

##### Biology.

Seasonality and daily activity time are in the same as in *G. serrator* (see there), but the species seems to spend more time under shrubs. *Graphipterus multiguttatus* prefers stabilized and semi-stabilized sand with high vegetation. The population densities in the sympatric areas of the distribution ranges are lower than those for *G. serrator*. The beetles dig burrows between the hard crust layer and the soft sand, sometimes close to the dwarf-shrubs. Frequently, the openings do not collapse or become covered by sand. The beetles sometimes close the openings with sand from inside. Diet, intraspecific behavior including copulation and the chirping sounds produced by the stridulatory structure, are same as in *G. serrator*.

##### Scraping record.

In comparison to the co-occurring *G. serrator*, the scraping spectrograms of *G. multiguttatus* show clear differences in pulse interval as well as in the sound pressure level (Fig. 10b).

#### Graphipterus peletieri

Taxon classificationAnimaliaColeopteraCarabidae

Laporte de Castelnau, 1840: 58, stat. rest.

Figs 3e , 9i , 17 , 24b

Graphipterus lepeletieri Alluaud, 1926: 17 (Tissaf)
Graphipterus discipennis Chevrolat [Unpublished name]

##### Types.

Holotype: ♂ (Blue label, black handwritten): <*Pletieri*. Chevrolat./Oran. D.S Fargeau>. (White label, black typewritten): <P. Bedel/Visit 1905>. (White label with brown margin, brown typewritten): <EX Musaeo/Chaudoir>. (Red label, black typewritten): <TYPE>. Deposited in NHMB, Chaudoir collection [examined].

##### Diagnosis.

Predominantly dark, medium-sized species with 18-24 small, mostly rounded white spots on elytra, four usually short marginal extensions. Median lobe of aedeagus with ventral, short, unbent tip.

##### Comparisons.

*Graphipterus peletieri* resembles *G. luctuosus* (see comparisons in *G. luctuosus*).

##### Description.

BL male: 13.9–14.8 mm, average 14.3 ± 0.4 mm; BL female: 11.5–16.1 mm, average 13.6 ± 1.8 mm.

*Head* medium; HW/PW: 0.76; EYL: 1–1.5 mm; EYL/EL: 0.17. Mentum with usually three teeth (Fig. 3d). Frontal ridge absent. In male, apical white frons stripes slenderer than exposed frons (cf. Fig. 4a).

*Pronotum* strongly cordiform, PL/PW: 0.63; BPW/PW: 0.6; posteromedially concave and without white margin; white lateral margin as wide as antennomere I long.

*Elytra* oval, humeri rounded; EL: 7.4–9.1 mm, average 8; EW: 5.7–7.9 mm, average 7.0 mm; EL/EW: 1.15. Elytra with brown scales, disc of elytra visible between scales (cf. Fig. 6b). White lateral margin wide as half antennomere I long and with four extensions; extensions often constricted; extension I elongated, shorter than extension II; sutural gap of white posterior margin wider than lateral margin. Disc usually with 18–24 mostly rounded spots; anterior pair of spots slightly elongate, as wide as extension I, lateral spots rounded, adjacent or sometimes fused to extension II, posterior pair of spots rounded, slightly larger than others, located toward suture. Lateral cross section quite flat. Apical sinuation developed, apex slightly protruded, strongly rounded (cf. Fig. 7b). Suture inconspicuous.

*Legs* long; MTIL: 3.8–5.3 mm, average 4.8 mm; El/MTIL: 1.7. Metatibial secondary spur brown. MTAL: 2.8–4.0 mm, average 3.5 mm; MTAL/MTIL: 0.7 (all other species of the *G. serrator* group El/MTIL: 0.8). Claws of hind legs brown at base.

Median lobe of *aedeagus* with ventrally short, not bent tip (Fig. 9i).

##### Habitat.

Unknown.

##### Co-occurring species.

*Graphipterus peletieri* lives in sympatry with five other species in north-west Algeria: *G. luctuosus*, *G. rotundatus*, *G. valdanii*, *G. stagonopsis* sp. n., and *G. piniamitaii* sp. n.

##### Distribution.

North-west Algeria and north Morocco (Fig. 17).

##### Conservation.

The species does not seem to be threatened as it has a wide distribution range that appears to be mostly not strongly affected by human activities.

##### Comments.

Alluaud (1926) initially erroneously named this species *Graphopterus lepeletieri* and since then this spelling has commonly been used by many authors (e.g., Basilewsky 1977). *Graphipterus luctuosus* was ranked as a subspecies of *G. peletieri* by Basilewsky (1977), but as a “good” species by Lorenz (2005) and Huber and Marggi (2017).

#### Graphipterus piniamitaii

Taxon classificationAnimaliaColeopteraCarabidae

Renan & Freidberg sp. n.

http://zoobank.org/7B3CE213-D0D6-4083-AA8E-109E72094EEE

**Figure 13.**
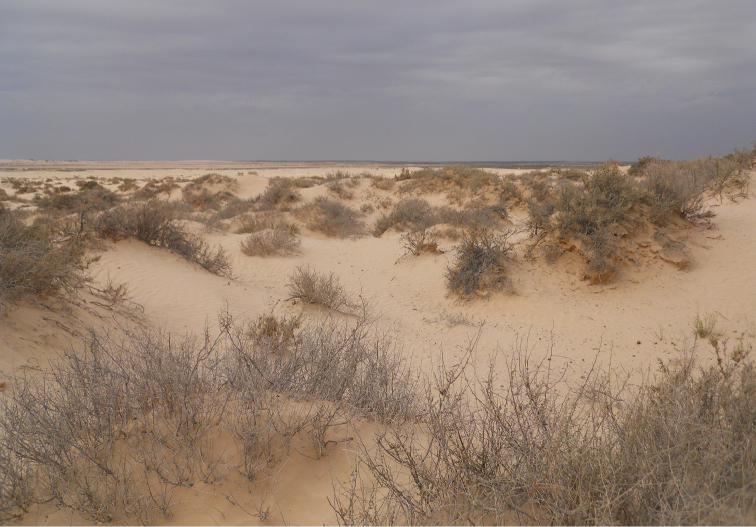
Habitat of *Graphipterus piniamitaii* sp. n.: Shifting sand dunes with vegetated Nebka hills, Kebili, Tunisia.

Figs 9j , 13 , 18 , 25a

##### Types.

Holotype: ♂ (White label, black handwritten): <Kebili>. (White label, black typewritten): <Ex Museo/L. Vibert>. (ae). Deposited in NHMB, general collection.

**Paratypes**. (20♂, 4♀). El Hammama, Tunis: (Gabès), I. 1889, Alluaud (♂) (ZMUC); Gafsa Tunis, Vibert Lyon (♂) (NMP). Kebilli, Tunis: 1906, EX Call. Maindron M., Call G. Babault 1930 (♂ae) (NHMB, General collection); 1950, Cobos Sa’nchez, (uc) (♂) (NHMB, Negre collection); L. Vibert, Ex Musaeo (♂ae) (MRAC); Call. Mus Congo, Col. P. Basilewsky (5♂) (RMRAC); Tunisia, Kebili 15 km N.W, 17.III. 1986, Zool. Mus. Copenhagen Exp. (3♂) (ZMUC); Kebili 2 km s, W. Ziegler, 30 m, Dünen, 5.3.2012, (♂, 2♀) (DWC, CAB); Douz, south Tunisia, Zaafrane (Sahara), 02.04.1992 (♂). S. Tunisia (Kebili), Zaafrane, 12 km SW Douz, 21.IV.2007 M. Liebscher (♀); S. Tunisia (Kebili), Zaafrane, 12 km SW Douz, 21.IV.2007 M. Liebscher (♀); C. Tunisia, 2 km E. Kairouan, 23.4.2005, M. Liebscher Sammlung (♂,♀) (DWC). Oasis Gafsa: Tunis, B v. Bodemeyer (♂) (DEI Muncheberg Call- 01314); B v. Bodemeyer, O. Leonhard, (uc) (♂ae) (DEI Muncheberg Call- 01315); B v. Bodemeyer, O. Leonhard (♂) (DEI Muncheberg Call- 01316).

##### Diagnosis.

Medium-sized species with usually 24 white large rounded and elongated spots on elytra; posterior discal spots slightly larger than other spots; six marginal extensions (Fig. 25a). Median lobe of aedeagus with slightly bent tip.

##### Comparisons.

*Graphipterus piniamitaii* sp. n. is easily distinguished from all other species of the group by its large white spots on the elytra. The new species resembles *G. mauretensis* sp. n. (see comparisons in *G. mauretensis* sp. n.).

##### Description.

BL male: 15.5–19.8 mm, average 17.5 ± 2.1 mm; BL female: 17–17.9 mm, average 17.5 ± 0.3 mm.

*Head* medium; HW/PW: 0.76; EYL: 1.5–1.8 mm; EYL/EL: 0.17. Mentum with two or three teeth. Frontal ridge absent. In male, apical white frons stripes wider than exposed frons (cf. Fig. 4b).

*Pronotum* cordiform; PL/PW: 0.63; BPW/PW: 0.66; posteromedially concave and without white margin; white lateral margin as wide antennomere I long.

*Elytra* oval, humeri rounded, but slightly protruding; EL: 8.2–11.0 mm, average 9.4 mm; EW: 6.5–9.2 mm, average 8.1 mm; EL/EW: 1.2. Lateral cross section quite flat. Suture conspicuous. Black scales dense, disc not visible between scales (cf. Fig. 6a). White lateral margin nearly as wide as 1½ antennomere I long and with six extensions; extension I triangular, slightly elongated and posteriori oriented; extensions II and III frequently constricted at base, usually wider than lateral margin. White posterior margin as wide as lateral margin or wider, not becoming narrower towards the suture; gap at suture smaller than lateral margin. Disc usually with 24 rounded to elongate, moderate large spots; anterior spot elongated, as wide as extension I, posterior discal spots slightly larger than other spots, series of six elongated spots along suture, sometimes fused to each other; posterior discal spots larger than other spots. Apical sinuation strongly developed, apex protruded, almost rectangular, only slightly rounded at most distant tip (cf. Fig. 7a).

*Legs* long; MTIL: 5.3–6.6 mm, average 6.1 mm; El/MTIL: 1.54. Metatibial secondary spur brown at base, MTAL: 4–5.7.0 mm, average 4.9 mm; MTAL/MTIL: 0.8. Claws of hind legs brown at base.

Median lobe of *aedeagus* with slightly bent tip (Fig. 9j).

##### Etymology.

The species is dedicated to Pinchas (Pini) Amitai, an inspiring entomologist and mentor who wrote the first Hebrew photographed insect guide.

##### Habitat.

The species dwells in the vicinity of Kebili on intensively grazed dunes, together with Anthia (Thermophilum) sexmaculata (Fabricius, 1787) and *A. venator* (Fabricius, 1792) (Fig. 13). The dunes have a diverse vegetation of shrubs and dwarf-shrubs.

##### Co-occurring species.

*Graphipterus piniamitaii* lives in sympatry in Kebili and Gabès region in Tunisia with *G. luctuosus*, *G. peletieri*, and *G. heydeni*.

##### Distribution.

Restricted to Central Tunisia, from the vicinity of Kebili to Gabès (Fig. 18).

##### Conservation.

The species does not seem to be endangered, as the preferred habitat is not strongly affected by human activities.

#### Graphipterus reymondi

Taxon classificationAnimaliaColeopteraCarabidae

Antoine, 1953: 208 stat. n.

Figs 6b , 9k , 16 , 25b

##### Types.

Holotype: ♂ (White label, brown handwritten): <*Reymondi m.*/ (same label, black typewritten): Antoine det.>. (White label, black handwritten): <Inhamid/ Sahara septe./(Reymond)>. (Red label, black handwritten): <HOLOTYPE>. Deposited in NHMB, General collection, box 31[examined].

##### Diagnosis.

Large species with 20-24 isolated white round spots on elytra, six marginal extensions, extension II short, almost triangular. Humeri very narrowed, maximum width of elytra at interior rear third. The discal elytra pattern comprises a group of 8–12 elongated spots in an order parallel to the suture. Median lobe of aedeagus with ventrally bent tip.

##### Comparisons.

*Graphipterus reymondi* resembles *G. sharonae* sp. n., from which it differs mainly by mentum and humeri morphology, pattern, color and morphology of elytra (see full comparisons under *G. sharonae* sp. n.). *Graphipterus reymondi* resembles also *G. stagonopsis* sp. n., from which it differs mainly by mentum morphology, pattern, and morphology of elytra, and color of claws and spurs (see full comparisons under *G. stagonopsis* sp. n.).

##### Description.

BL male: 17–18, average 17.6 ± 0.4 mm; BL female: 17.4–21.4, average 19.3 ±2 mm.

*Head* medium; HW/PW: 0.76; EYL: 1.6–1.8 mm; EYL/EL: 0.17. Mentum with three teeth (cf. Fig. 3d). Frontal ridge absent. In male, apical white frons stripes wider than exposed frons (Fig. 4b).

*Pronotum* wide; PL/PW: 0.72; BPW/PW: 0.63; posteromedially concave and without white margin; white lateral margin as wide as antennomere I long.

*Elytra* with strongly narrowed humeri; EL: 9.4–10.3 mm, average 9.75 mm; EW: 8.0–8.5, average 8.3 mm; EL/EW: 1.2. Lateral cross section convex. Suture conspicuous. Scales brown, disc visible between them (Fig. 6b). White lateral margin nearly as wide as antennomere I long and with six extensions; extension I triangular with rounded tip, slightly more elongated than in *G. serrator*, wider and shorter than extensionII; the latter one elongated, at third quarter of elytra. White posterior margin commonly slightly wider than lateral margin, gap at suture smaller than lateral margin, usually with a small, indistinct tip anteriorly. Disc usually with 20–24 (rarely 18), mainly rounded spots; anterior pair of spots rounded, wide as extension I, usually smaller than posterior spots, but larger than spots on central disc; central disc spots usually asymmetrically smeared; posterior pair of spots rounded; one or two small additional spots adjacent laterally to the posterior ones. Apical sinuation strongly developed, apex protruded, almost rectangular, only slightly rounded at most distant tip (Fig. 7a).

*Legs* long; MTIL: 5.8–6.1 mm, average 5.9 mm; El/MTIL: 1.6. Metatibial secondary spur brown. MTAL length: 4.2–5, average 4.7 mm; MTAL/MTIL: 0.8. Claws of hind legs black at base.

Median lobe of *aedeagus* with ventrally bent tip (Fig. 9k).

##### Habitat.

Unknown.

##### Co-occurring species.

No co-occurring species.

##### Distribution.

Morocco (Fig. 16).

##### Conservation.

Unknown.

#### Graphipterus rotundatus

Taxon classificationAnimaliaColeopteraCarabidae

Klug, 1832: 7, stat. rest.

Figs 7a , 9l , 18 , 26a

##### Types.

Lectotype: ♀ (blue label, black handwritten): <rotundatus/Klug*/x.118-21./ Bir Hamam El Eherenberg> (White label, black handwritten): <Zwischen Bir-Lebuck/and Bir Hamam/(Libye)> (White label, black typewritten): <Type> (White label, black typewritten): <Hist. –Coll. (Coleoptera)/Nr. 1299/ *Graphipterus rotundatus*/ Klug*/Bir Hamam El Eherenberg/Zool. Mus. Berlin>. Deposited in ZMHB [examined]. Paralectotype: ♂ (Red label, black typewritten): <Type> (White label, black handwritten): <1299> (white label, black typewritten): <Hist.–Coll. (Coleoptera)/Nr. 1299/*Graphipterus rotundatus*/Klug*/Bir Hamam El Eherenberg/Zool. Mus. Berlin>. Deposited in ZMHB [examined].

##### Diagnosis.

Small species with large distribution range, high variation in size (15–19 mm) and variation in elytra pattern (4–6 extensions, 16–22 spots). posterior discal spots larger than other spots; six spots usually forming an arc pattern anterior and lateral to posterior spots; Median lobe of aedeagus with short, slightly bent tip.

##### Comparisons.

*Graphipterus rotundatus* resembles *G. multiguttatus* (see comparisons in *G. multiguttatus*) and *G. luctuosus* (see comparisons in *G. luctuosus*).

##### Description.

BL male: 15.0–19.0 mm, average 17.4 ± 1.5 mm; BL female: 15.4–17.1 mm, average 16.1 ± 1.3 mm.

*Head* slender; HW /PW: 0.72; EYL: 1.4–1.7 mm; EYL/EL: 0.16. Mentum with 2–3 teeth. Frontal ridge slightly developed. In male, apical white frons stripes wider than exposed frons (Fig. 4b).

*Pronotum* cordiform; PL/PW: 0.65; BPW/PW: 0.69; posteromedially concave and without white margin; white lateral margin as wide antonomer 1 long.

*Elytra* oval, humeri rounded; EL: 8.9–11.0 mm, average 9.7 mm; EW: 7.0–8.7 mm, average 7.8 mm; EL/EW: 1.25. Lateral cross section quite flat. Dense black scales, disc not visible between scales (Fig. 6a). White lateral margin nearly as wide as half antennomere I long and with six, sometimes fouor extensions; extension I triangular to slightly elongated; extension II absent or only weakly developed, rarely fused with lateral disc spot. White posterior margin becomes narrower towards the suture, gap at suture smaller than lateral margin. Disc usually with 18, sometimes 16 or 22 rounded to weakly elongate spots; anterior spot slightly elongated, wide as extension I, six spots usually forming an arc pattern anterior and lateral to posterior rounded, larger spots. Apical sinuation strongly developed, apex protruded, almost rectangular, only slightly rounded at most distant tip (Fig. 7a). Suture conspicuous.

*Legs* long; MTIL: 4.3–5.2 mm, average 4.7 mm; El/MTIL: 1.63. Metatibial secondary spur dark at base, MTAL: 5.4–6.9 mm, average 6 mm; MTAL/MTIL: 0.8. Claws of hind legs brown at base.

Median lobe of *aedeagus* with slightly bent tip (Fig. 9l).

##### Habitat.

Unknown.

##### Co-occurring species.

*Graphippterus rotundatus* lives in sympatry with G. *luctuosus, G. peletieri*, and *G. valdanii* in Algeria and Tunisia.

##### Distribution.

Algeria, Tunisia, and the coastal region of west Libya (Fig. 18).

##### Conservation.

The species does not seem to be endangered as it has a wide distribution range which is not strongly affected by human activities.

##### Comments.

On the label of the *Graphipterus rotundatus* type, “Libye” is written; however, as far as it is known, C.G. Ehrenberg never succeeded in reaching Libya (Baker, 1997). There is only a very small chance that any other entomologist had collected *Graphipterus* in Lybia earlier than 1830.

##### Biology.

The three larval stages develop during the summer inside ant nests. The first larval instar is nearly 4 mm long and creeps into nests of large ant species, digs there a chamber, preys on the ant’s brood and pupates within the nest. When the first larval instar tries to enter nests of small ants, it is attacked by the ants (Paarmann 1985; Dinter et al. 2002). The larval instars have a mandibular suctorial tube to suck hemolymph from their prey (Brandmayr 1994a, 1994b). Four specimens from the species studied by Wilfried Paarmann, Pietro Brandmayr, and their co-workers were examined; the material belongs to *G. rotundatus* and not to *G. serrator* as noted in their publications.

#### Graphipterus serrator

Taxon classificationAnimaliaColeopteraCarabidae

(Forskål, 1775): 77

Figs 1 , 3f , 4b , 4c , 5 , 6a , 7 , 9m , 10a , 14 , 19 , 26b , 28b, c, d

Carabus serrator Forskål, 1775: 77 (Aegypten)
Carabus variegatus Fabricius, 1781: 501 (Orient)
Carabus variegatus Fabricius, 1792: 147 (Orient)
Graphipterus serrator lobatus Alfieri, 1976: 15 [unavailable name]
Graphipterus serrator sexguttatus Alfieri, 1976: 15 [unavailable name]

##### Type material of *Carabus serrator*.

Holotype: ♀ (White label with blue margin, black handwritten): <*Graphipterus* Latr./*serrator* Forsk./Aegypten>. Deposited in ZMUC [examined].

##### Type material of *Carabus variegatus*.

Holotype: gender unknown (only fragments of a beetle preserved). (White label with black margin, black handwritten): < variegatus/ 824>. Deposited in ZMK [examined] (Fig. 28d).

##### Diagnosis.

Large species with 10–12 isolated white round spots on elytra: anterior and posterior discal spots larger than other spots, six smaller spots near suture form circular pattern on disc; four white marginal extensions present, extension I triangular. Median lobe of aedeagus with ventrally bent tip.

##### Comparisons.

*Graphipterus serrator* resembles *G. valdanii* from which it differs mainly by the following characters: *G. serrator*: mentum with three teeth, mid tooth shallow; PL/PW (0.72); BPW/HW (0.8); EL/EW rounded (1.18); elytra lateral margin wide as antennomere I long; Claws of hind legs dark. *G. valdanii*: mentum with three teeth, merges shallow and mid tooth bolt; PL/PW (0.64); BPW/HW (1); EL/EW elongated (1.3); elytra lateral margin wide as half antennomere I long; Claws of hind legs brown.

##### Description.

BL male: 17–18 mm, average 17.6 ± 0.4 mm; BL female: 17.4–21.4 mm, average 19.3 ± 2 mm.

*Head* medium; HW/PW: 0.76; EYL: 1.6–1.8.0 mm; EYL/EL: 0.16. Mentum with three teeth, mid tooth shallow (Fig. 3f). Frontal ridge absent. In male, Apical white frons stripes slenderer than exposed frons (Fig. 4b). Pronotum wide; PL/PW: 0.58; BPW/PW: 0.65; posteromedially concave and without white margins; white lateral margin as wide as antennomere I long.

*Elytra* oval, humeri rounded; EL: 9.3–11.3 mm, average 10.3 mm; EW: 7.0–9.8 mm, average 8.4 mm; EL/EW: 1.2. Lateral cross section convex. Elytra with dense black scales, disc of elytra not visible between scales (Fig. 6a). White lateral margin nearly as wide as antennomere I long and with four extensions; extension I triangular with rounded angels, margin of elytra wider and shorter than extension II; the latter one elongated; at third quarter of elytra, imaginary line connecting the medial ends of the extension I and I parallel to the suture. White posterior margin forming gap at suture which is wider than lateral margin. Disc usually with 10, sometimes 12 round spots; anterior pair of spots circular to slightly elongate, narrower than extension I, larger than the six central spots forming a circular pattern; anterior and posterior pair of spots circular rounded, larger than other spots; small additional spots frequently present laterally to the posterior spots. Apical sinuation strongly developed, apex protruded, almost rectangular, only slightly rounded at most distant tip (Fig. 7a). Suture inconspicuous.

*Legs* long; MTIL: 4.8–7.4 mm, average 6.1 mm; El/MTIL: 1.7. Metatibial secondary spur dark. MTAL: 4.0–5.3 mm, average 4.6 mm; MTAL/MTIL: 0.8. Claws of hind legs black at base. Median lobe of aedeagus with ventrally bent tip (Fig. 9m).

##### Habitat.

**Figure 14.**
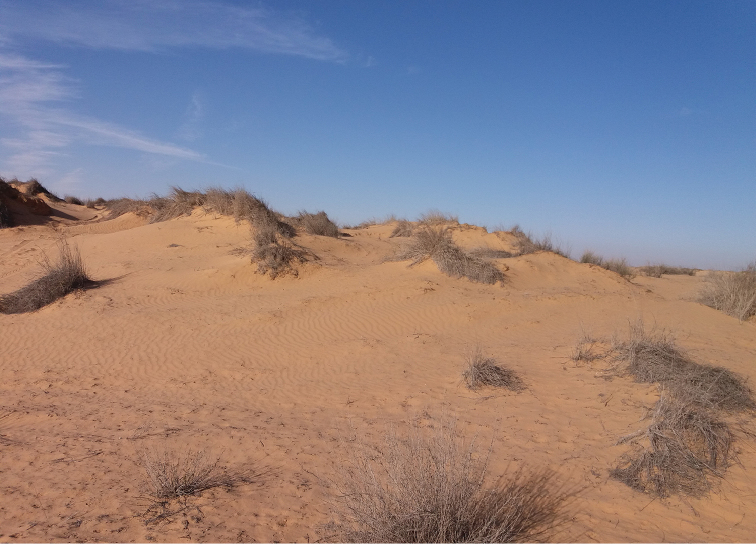
Habitat of *Graphipterus serrator*: Shifting sand dunes in the Western Negev Sands, Israel.

Very common in arid sandy habitats, it shows a significant habitat preference for the crest of shifting sand dunes (Fig. 14). It avoids stabilized interdunes and half-stabilized dune slopes (Renan et al. 2011). The sandy habitat in the western Negev sand dunes is poor in perennial woody plants with maximal coverage of 10–15% (Perry 2008; Siegal et al. 2013). The dominant perennial plants are *Retama raetam* (Fabaceae) and *Stipagrostis scoparia* (Poaceae).

##### Co-occurring species.

*Graphipterus serrator* lives in sympatry with *G. multiguttatus* in Egypt and Israel.

##### Distribution.

North-east Egypt (incl. Sinai) and Israel. In Israel it is restricted to the western Negev sand dunes (Fig. 19).

##### Conservation.

The sand dunes in the western Negev suffer from two major threats: agricultural development that has caused a significant loss of the sands’ range (Ben David and Avni 2013), and a stabilizing process of the shifting sand resulting from a bio-crust (Kidron and Abeliovich 2009). In the Sinai Peninsula, a lack of shrubs as a result of overgrazing threats the population.

##### Comments.

The female holotype of *Carabus serrator* has been considered lost (Basilewsky 1977), but it was recently found by us in ZMUC (Fig. 28b–c). After studying the type material, we agree with Hůrka (2003), Lorenz (2005) and Huber and Marggi (2017), that *variegatus* (Fabricius 1792) falls within the morphological variability of *serrator*. Therefore *variegatus* is confirmed as a junior synonym of *serrator* (Fig. 28d). *Graphipterus serrator lobatus* and *G. serrator sexmaculatus* were considered by Alfieri (1976) as variations of *G. serrator*. Following the ICZN (1999, Article 45.6.3), a taxon that is described as a variation after 1960 is not valid. Moreover, no holotype has been designated. Therefore, both *lobatus* Alfieri and *sexmaculatus* Alfieri are not available names. One specimen from the western Negev sands was found with intermediate characters of *G. serrator serrator* and *G. multiguttatus*, and this specimen seems to be a hybrid between them: ♂ Israel, Holot Agur, May 2012, leg. I. Renan.

##### Biology.

Adults emerge immediately after the first significant rainfall and inhabit sandy dunes or sand and loess plains and edges of salt lakes. In the spring following an average rainy winter, the species can densely populate the dunes (one observer can locate up to 40 individuals within one hour). Their diet is based mainly on ants and occasionally on other small insects, as well as on dead insects and dead reptiles. Activity is limited by temperature: it begins at a soil temperature of approximetly 18 °C, and ceases at a soil temperature of approximetly 39 °C. By moving between sun-exposed microhabitats and the shadow of dwarf-shrubs can prolong the activity period. Strong wind halts activity due to the beetle's sensitivity to dehydration. Some activity also occurs in the afternoon, but it is significantly lower than in the morning peak hours.

Prior to commencing inactivity, the beetle digs a short burrow with a narrow elliptic cross-section into the dune’s slope. The digging is performed mainly with the hind legs and secondarily with the middle legs. The well-developed, spoon-shaped metatibial spurs (see fig. 4a in Assmann et al. 2015) seem to function as a shovel. The burrow’s opening usually collapses behind the beetle or is covered by shifting sand. In the burrow, a few centimeters below the sand surface, the beetle is relatively protected from predation and can probably still detect the outside temperature and light conditions. In enclosure experiments with individual markings and variation in population density, one of us found that even during the peak activity season, most of the specimens spend most of the days without displaying epigeic activity. An encounter between two individuals of any gender immediately develops into a short, hasty, bite battle and the escape of the loser. In some regions, shade is a limited resource and the battle occurs mainly under bushes and dwarf-shrubs. An encounter between male and female starts with an aggressive fight. The persistent male will then mount the back of the female. His forelegs grasp the female between the basal part of the pronotum and the elytral humeri, while the female tries to grab the male with her hind legs. The copulation lasts for approximately 30 minutes and occurs mostly beneath perennial vegetation. During the fight, the beetles stridulate. This sound is produced when the beetles are threatened by other individuals or by potential predators (Renan unpublished data, based on field observations and arena experiments).

##### Scraping record.

Comparing *G. serrator*’s scraping spectrograms with those from its co-occurring species, *G. multiguttatus*, reveals clear differences in pulse intervals as well as in the sound pressure level (Fig. 10).

#### Graphipterus sharonae

Taxon classificationAnimaliaColeopteraCarabidae

Renan & Assmann sp. n.

http://zoobank.org/64BF5A31-99ED-4C75-A88F-3FB3144618B1

**Figure 15.**
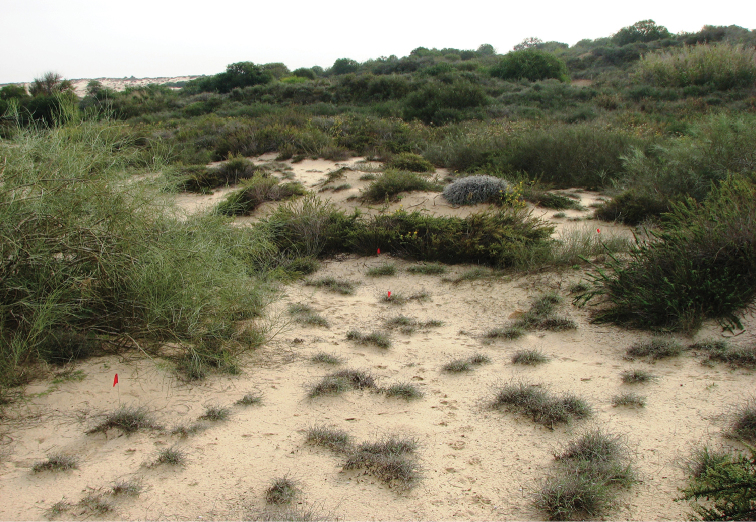
Habitat of *Graphipterus sharonae* sp. n.: Stabilized sand dunes with rich vegetation. Nizzanim, Israel.

Figs 9n , 15 , 19 , 27a

##### Types.

Holotype, ♂ (White labe, black typewritten): <51780 ISRAEL/ Karmiya N.P/ 7.4.2011/ I. Renan>. (red label): <Holotype>(ae). Deposited in SMNHTAU [examined].

Paratypes: (79♂, 70♀): All material collected in Israel. Ashdod: 6.V.2015, I. Renan (7♂, 14♀); 5.XII.2014, I. Renan (6♂, 3♀); 16.III.2011, (♂) (CAB); 3.IV.1998, H. Ackerman (♂) (SMNHTAU); 16.III.2011 leg. Th. Assmann, (♂,♀), W. Starke leg. (♂,♀) (CAB). Ashkelon [Ashqelon]: 7.IV.2017, I. Renan (6♂, 4♀) (SMNHTAU). Avshalom: 24.III.2012, M. Bologna (2♂) (AVTC). Ayalon: 1.IV.1943 (♂) (KCE). Bat Yam: 14.III.1940, Bytinski- Salz (3♂); 24.III.1940, Bytinski- Salz (2♂); 23.IV.1959, J. Wahrman (2♂, 2♀) (SMNHTAU). Bene’ Berack [Bene Beraq]: 26.II.1954 (♂) (SMNHTAU). ‘En Sarid: 22.IV.2015, I. Renan (2♀) (SMNHTAU). Holon: 14. IV.1981, A. Freidberg (♂) (SMNHTAU). Jaffa [Yafo]: 21.I.1900 (♀) (BMNH). Jaffa-Rehoboth [Rehovot]: 14.VII.1913, S.G.J. Aharoni (♂) (RMRAC). Karmiyya N.P: 07.IV.2011, I. Renan (4♂, 2♀) (SMNHTAU). MiqWeYisra’el: 14. IV.1934, F.S. Bodenheimer (♂, 3♀); 11.IV.1946, J. Wahrman (♀); 20. IV.1934, F.S. Bodenheimer (♂) (SMNHTAU). Nachalat Jischack, Palestina [Tel Aviv, Nahalat Yizhaq], 5.VI.1942, Housk (4♂) (NMP). Netanya: III.-IV. 1996, R. Rod (♀) (DWC); 15.II.1955, S. Nothiltz (♂); 11. IV.1957, J. Machlis (♂, ♀); 03.V.1997, R. Hoffman (♂, ♀); 04. IV.2010, I. Renan (5♂, 1ae, ♀) (SMNHTAU); III.IV.1996, leg. R. Rod (♀) (CWD); III.2016, leg. Th. Assmann (3♂, 4♀) (CAB). Nizzanim N.P: 29. IV.2015, Renan I. (14♂, 10♀); 19.V.2009, I. Renan (3♂,2ae, ♀); 15.V.2009, I. Renan (5♂, 2ae, 4♀,) 7.4.2011, I. Renan (♂ae) (SMNHTAU). 22.III.2012, M. Bologna (♂) (AVTC). 25.II.2009, L. Friedman (♀) (BMNH); 07.VI.2007, leg. J. Buse (♀) (CAB). Palmahim: 25.III.1978, Tedeschi (3♂, 1♀) (AVTC). Porat: 22.I.2015, I. Renan (♂); 09.IV.2014, I. Renan (5♂); 19.IV.2015, I. Renan (2♂); 22.IV.2015, I. Renan (8♂, 8♀) (SMNHTAU). Ra’ananna: 11.IV.1947, Bytinski-Salz (♂) (SMNHTAU). 13.VI.1940, Bytinski-Salz (♀) (SMNHTAU). Rafha [Rafiah]: (♂) (ae) (BMNH). Rishon Leziyyon: 17.III.2003, M. Yogev (♂) (BMNH); 10.III.1942, Bytinski-Salz (♀); 29.VI.1979, D. Furth (♂); 1.III.1938 (♂) (SMNHTAU). Tel Aviv: 11.I.1900 (♂) (BMNH); 2.I.1900 (♂) (KCE). Ziqim N.P: 4.VI.2015, I. Renan (2♂, 4♀); 5.V.2015, I. Renan (♂, ♀); 7.IV.2011, I. Renan (2♂, 1♀) (SMNHTAU).

##### Diagnosis.

Medium-sized species with 12–18 white elytral spots; the anterior and central ones usually elongated, the posterior ones rounded; six marginal extensions, extension II triangular. Median lobe of aedeagus with bent tip.

##### Comparisons.

*Graphipterus sharonae* sp. n. resembles *G. multiguttatus*, from which it differs mainly by the following characters: *G. sharonae* average body length 13mm; extension slightly elongated 1; median lobe of aedeagus short, unbent tip. *G. multiguttatus* average body length 17.05 mm; extension I triangular; median lobe of aedeagus with bent tip. *Graphipterus sharonae* resembles also *G. reymondi*, from which it differs mainly by the following characters: *G. sharonae* sp. n. mentum with two teeth, humeri rounded, 12–18 spots on elytra, widest line of elytra located at middle, elytra disc not seen, and elytra scales black while *G. reymondi* has the mentum with three teeth, humeri narrowed, 20–24 spots on elytra, widest line of elytra located at interior rear third, elytra disc seen, and elytra scales brown.

##### Description.

BL male: 15.0–18.0 mm, average 16.5 ± 0.8 mm; BL female: 16.0–193 mm., average 17.6 ± 0.8 mm;

*Head* medium; HW/PW: 0.74 mm; EYL: 1.2–1.7 mm; EYL/EL: 0.15. Mentum with two teeth and shallow depression between (cf. Fig. 3b). Frontal ridge slightly developed. In male, apical white frons stripes wider than exposed frons (Fig. 4b).

*Pronotum* cordiform; PL/PW: 0.66; BPW/PW: 0.66; posteromedially concave and without white margin; white lateral margin as wide as antennomere I long.

*Elytra* oval, humeri rounded; EL: 8.1–10.3 mm, average 9.2 mm; EW: 6.2–8.8 mm, average 7.8 mm, (EL/EW: 1.3). Lateral cross section flat. Dense black scales, disc not visible between scales (Fig. 6a). White lateral margin as wide as half antennomere I long and with six extensions; extension I triangular with rounded angels, as wide as or wider than elytra margin, wider and shorter than extension III; extension II smaller and usually shorter than two other ones; extension III often constricted at base. White posterior margin almost continuously rounded, only slightly becoming narrower, gap at suture smaller than lateral margin. Disc with 14, sometimes 12 or 18 spots; most anterior pair of spots slightly elongate to rounded, usually wide as extension I, second anterior pair of spots strongly elongate, nearly two times as long as wide; the two lateral pairs of spots rounded, adjacent or sometimes fused to extension II; the tow to four posterior pairs of spots rounded; the medial, most posterior pair of spots larger than all other spots; the outer most posterior pair of spots much smaller than the latter one. Apical sinuation slightly developed to straight, apex not protuberant, broadly rounded, especially on the medial side (Fig. 7c). Suture conspicuous.

*Legs* long; MTIL: 4.9–6.1 mm, average 5.6 mm; El/MTIL: 1.7. Metatibial secondary spur brown. MTAL: 4.0–5.0 mm, average: 4.5 mm; MTAL/MTIL: 0.8. Claws of hind legs black at base.

Median lobe of *aedeagus* with bent tip (Fig. 9n).

##### Etymology.

The species is dedicated to Sharon Renan, biologist, conservationist, and the first author’s wife.

##### Habitat.

In sand dunes and on calcareous sandstone habitats along the coast. Low, mostly vegetated and stabilized sand dunes are the preferred habitat (Ramot 2008). Individuals are active as far as 50 meters from the shoreline, but seem to be more common further inland. The average annual rainfall in the coastal plain is approxemetly 450 mm (I.M.S, 2016). The dominant perennials of the habitats in Israel are *Artemisia monosperma* and *Helianthemum stipulatum* (Fig. 15).

##### Co-occurring species.

No sympatrically occurring species.

##### Distribution.

Endemic to the Mediterranean coastal plains from north-east Sinai (El Arish) to central Israel south of Nahal Alexander (Fig. 19).

##### Conservation.

The coastal plain sand dunes of Israel form the largest part of the entire distribution range of *G. sharonae* sp n. As a result of land use changes and urbanization, less than 25% of the Israel sandy habitats remain and a further decline can be expected. In addition, the remaining dune habitats are under extreme anthropogenic disturbance and highly fragmented (Achiron-Frumkin et al. 2003). The following records are examples of sites that were populated by *G. sharonae* sp. n. in the past, but where their populations no longer exist: Kefar Bilu, Rehovot, Nes Ziyyona, Bat Yam, Holon, Tel Aviv, Ramat Gan, Bene Beraq, Ra’ananna, Yafo (based on SMNHTAU collection and the authors’ experience).

Despite having no precise data, the habitats in the Gaza Strip and north-eastern Egypt seem also to have declined as these areas feature a strong increase in human population density. In a faunistic survey of the ground beetles of the Sinai Peninsula, Abdel-Dayem et al. (2004) did not record *Graphipterus* from El-Arish, where it had been present nearly a century ago (records in London, cf. Schatzmayr 1936). El Surtasi et al. (2012) demonstrated the negative effect of urbanization on *G. serrator* population in Egypt. Both the restricted distribution range of the endemic species *G. sharonae* sp. n. and the decline in coastal sandy habitats threaten the long-term survival of the species.

##### Biology.

Seasonal and daily activity time, as well as diet, intraspecific behavior, including copulation and the chirping sounds produced by the stridulatory structure, are as in *G. serrator*. *Graphipterus sharonae* sp. n. prefers stabilized sands with high vegetation cover, and its population density is higher than that of *G. serrator*.

#### Graphipterus stagonopsis

Taxon classificationAnimaliaColeopteraCarabidae

Renan & Assmann sp. n.

http://zoobank.org/435E6626-47B0-4701-A57C-45E23959B7AD

Figs 18 , 27b

##### Types.

***Holotype.***♂ (White label, black handwritten): <Beni Abbes/23.III.48 F. Pierre>. (red label): <Holotype> (ae). Deposited in NHMB [examined].

***Paratypes.***(11♂, 3♀), NHMB (Colas collection): Gardhaia (Ghardaia), Sahara, G. Mahoux, 19.5.60 (2♂); Beni Abbes, 23.3.48, F. Paiu (2♂, 1- ae). (Negre collection): Beni Abbes, Sahara argelino, J. Mateu (3♀). (Antoine collection): Beni Abes, south Algerien (reymondi) (♂ae). ZMUC Algerie, Beni Abbes, 11.3.1984, Tilg. 4-12.1948, Tentens-Nielsen [*G. serrator* valdani Guer. P. Basilewsky 1985] ♂. NMP: Algeria, Igli, 12.IV. 1988, Igt. Kepler, 11/1988. Ex call. M. Dvorak, National Museum, Prague, Czech Republlic. MRAC: Aoulel el Arab Tidicelt Sahara Cen., J. Mateu (♂); Pozo zug (R.O.) Sa’hara espanol, J. Mateu (2♂); Oasis de la-Salah Tidikelt Sahara Cen, J. Mateu (3♂).

##### Diagnosis.

Large species with 16 white rounded to elongated spots on elytra, anterior and posterior pair of spots larger than others; six marginal extensions, extension I triangular, extension I and II elongated. Elytra widest at interior rear third, drop-like shape. Median lobe of aedeagus with slightly bent tip.

##### Comparisons.

*Graphipterus stagonopsis* sp. n. resembles *G. reymondi* from which it differs mainly by the following characters: G. *stagonopsis* sp. n.: mentum with two teeth; eight spots on elytra; scales of elytral disc brown; claws of hind legs and metatibial secondary spur dark . *G. reymondi*: mentum with three teeth; 10–12 spots on elytra; scales of elytral disc black; claws of hind legs and metatibial secondary spur brown .

##### Description.

BL male: 17.2–20.1 mm, average 18.8 ± 1 mm; BL female: 18.4–19.8 mm, average 18.9 ± 0.6 mm.

*Head* slender; HW/PW: 0.7; EYL 1.1–1.7 mm; EYL/EL: 0.16. Mentum with two teeth (Fig. 3b). Frontal ridge slightly developed. Male, apical white frons stripes wider than exposed frons (Fig. 4b).

*Pronotum* strongly cordiform; PL/PW: 0.66. BPW/PW: 0.64; posteromedially concave and without white margin; white lateral margin as wide as antennomere I long.

*Elytra* droplet-like, humeri strongly narrowed; EL: 9.1–11.1 mm, average 8.4 mm; EW: 7.5–9.0 mm, average 8.4 mm; EL/EW: 1.1–1.5. Lateral cross section convex. Dense black scales, disc not visible between them (Fig. 6a). White lateral margin nearly as half wide as antennomere I long and with six extensions; extension I triangular with rounded angels, as wide as lateral margin, posteriorly oriented; extension II small, often constricted at base, as wide as lateral margin; extension III large, elongated, posteriorly oriented. White posterior margin becomes narrower towards suture; gap at suture wider than lateral margin. Disc usually with 16 spots; anterior pair of spots elongate, as wide as extension I; posterior pair of spots rounded and larger than other ones; six spots forming arch pattern anterior and lateral to posterior rounded larger spots. Apical sinuation slightly developed to straight, apex not protuberant, broadly rounded, especially on the median side (Fig. 7c). Suture inconspicuous.

*Legs* long; MTIL: 6.0–7.0 mm, average 6.5 mm; El/MTIL: 1.6. Metatibial secondary spur black. MTAL: 4.7–5.3 mm, average 4.9 mm; MTAL/MTIL: 0.8. Claws of hind legs black at base.

Median lobe of *aedeagus* with bent tip (Fig. 9o).

##### Etymology.

The name is derived from ancient Greek (*σταγών*, *óψις*) and means ”drop-like” which refers to the shape of the elytra.

##### Habitat.

Unknown.

##### Co-occurring species.

*Graphipterus stagonopsis* lives in sympatry with *G. luctuosus, G. peletieri*, and *G. valdanii* in Ghardaia, Algeria.

##### Distribution.

Central and west Algeria (Fig. 18).

##### Conservation.

Unknown.

#### Graphipterus valdanii

Taxon classificationAnimaliaColeopteraCarabidae

Guérin-Méneville, 1859: 534, stat. rest.

Figs 3a , 17 , 28a

##### Types.

***Neotype*.** ♂ (White label, black handwritten): < Bou saada/ Oherthur R.>. (ae). Deposited in NHMB, General collection. (Red label, black typewritten): < Neotypus *Graphipterus valdanii* Guérin-Méneville, 1859/ des. I. Renan, 2018>.

***Neoparatypes***. NHMB (General collection): Baniou, Vibert L. (♂,♀); Bou saada, 1875, Oberthur R. (2♂); Bou saada, Oberthur R. (♂); Bou saada, Dr Martin (♂). (Negre collection): Algeria (♂); Bou Saada (♂); Bou Saada, Dr Martin (♀); BMNH: Bou-Saada, 1875, Oberthur R. (2♂). MRAC: Bou Saada, P. Basilewsky (♂); Bou saada, Dr Martin (♂). DEI: Bou Saada, O. Leonhard / Dr Martin (♂); ZMUC: Bou Saada, 28.4.1927 (♀); (uc) (♂);

##### Diagnosis.

Large species with 10–16 white round spots on elytra; anterior and posterior discal spots larger than other spots; four white marginal extensions, oval elytra, extension I triangular. Median lobe of aedeagus with bent tip.

##### Comparisons.

*Graphipterus valdanii* resembles *G. serrator* (see comparisons in *G. serrator*) and *G. heydeni* (see comparisons in *G. heydeni*).

##### Description.

BL male: 14.8–19.0 mm, average 17.1 ± 1.7 mm; BL female: 18.6–20.5 mm, average 18.6 ± 1.9 mm.

*Head* slender; HW/PW: 0.71; EYL: 1.4–1.8 mm; EYL/EL: 0.15. Mentum with mentum with two teeth as margin between them slightly convex in middle (Fig. 3c). Frontal ridge slightly developed. In male, apical white frons stripes slenderer than exposed frons (Fig. 4a).

*Pronotum* cordiform; PL/PW: 0.64; BPW/PW: 0.7; posteromedially concave and without white margin; white lateral margin as wide as antennomere I long.

*Elytra* oval, relatively elongated, humeri rounded; EL: 8.1–12 mm, average 10.6 mm; EW: 6.5–9.1 mm, average 8.0 mm; EL/EW: 1.3. Lateral cross section convex. Dense black scales, disc not seen between scales (Fig. 6a). White lateral margin as wide as half antennomere I long and with four extensions; extension I triangular with rounded angels, much wider than lateral margin, but shorter than extensionII; the latter one elongated, positioned at second third of elytra. Apical sinuation strongly sinuated, apex strongly protruded, forming almost a rectangular. White posterior margin evenly rounded, not becoming narrower towards the suture; gap at suture wider than lateral margin. Disc with (10–) 14 (–16) spots; anterior pair of spots rounded to slightly elongate, much smaller than extension I; anterior and posterior pair of spots round, larger than other spots; small additional spots located medially to extension II and laterally to posterior spots. Apical sinuation strongly developed, apex protruded, almost rectangular, only slightly rounded at most distant tip (Fig. 7a). Suture inconspicuous.

*Legs* long; MTIL: 4.9-7.0 mm, average 6.3 mm; El/MTIL: 1.7. Metatibial secondary spur dark. MTAL: 5.4-6.9 mm, average 6.0 mm; MTAL/MTIL: 0.72. Claws of hind legs brown at base.

Median lobe of *aedeagus* with bent tip (Fig. 9p).

##### Habitat.

Unknown.

##### Co-occurring species.

*Graphipterus valdanii* lives in sympatry with *G. peletieri*, *G. luctuosus*, *G. rotundatus*, and *G. stagonopsis* in Algeria.

##### Distribution.

The arid and semi-arid regions of north-east Algeria from Ghardaia, to Bou-Saada and Tebessa (Fig. 17).

**Figure 16.**
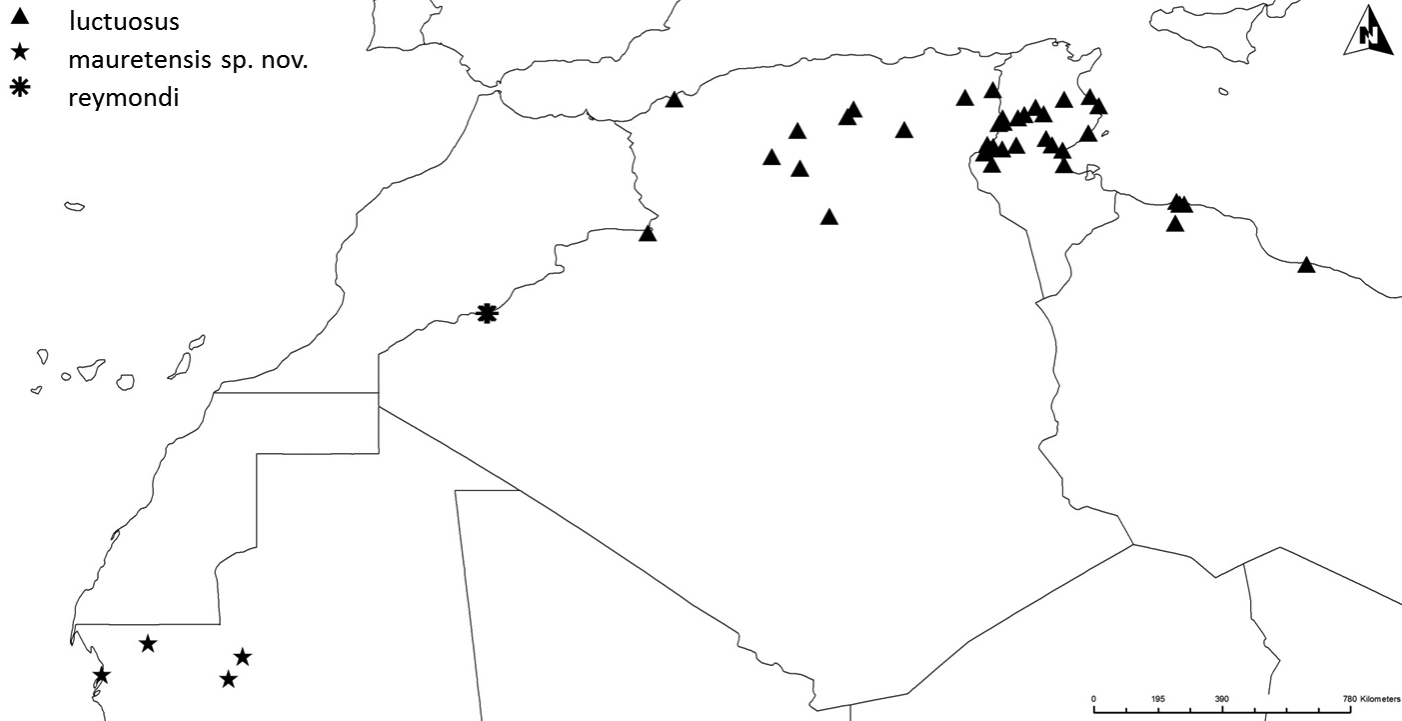
Distributional records of *G. luctuosus, G. mauretensis* sp. n., and *G. reymondi*.

**Figure 17.**
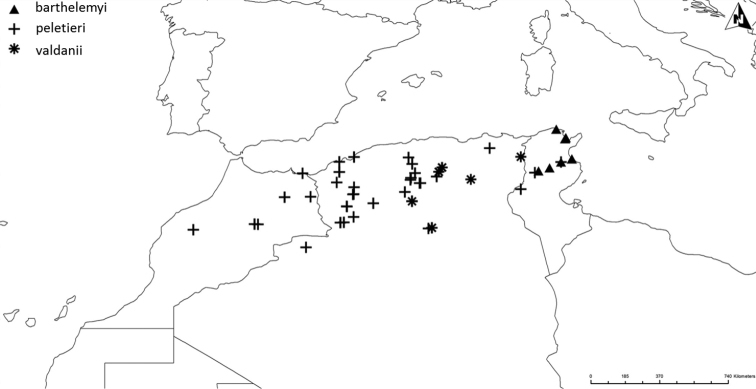
Distributional records of *G. barthelemyi*, *G. peletieri*, and *G. valdanii*.

**Figure 18.**
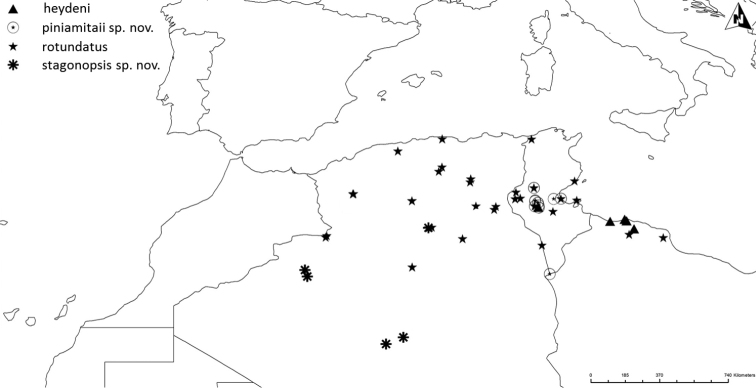
Distributional records of *G. heydeni*, *G. piniamitaii* sp. n., *G. rotundatus*, and *G. stagonopsis*, sp. n.

**Figure 19.**
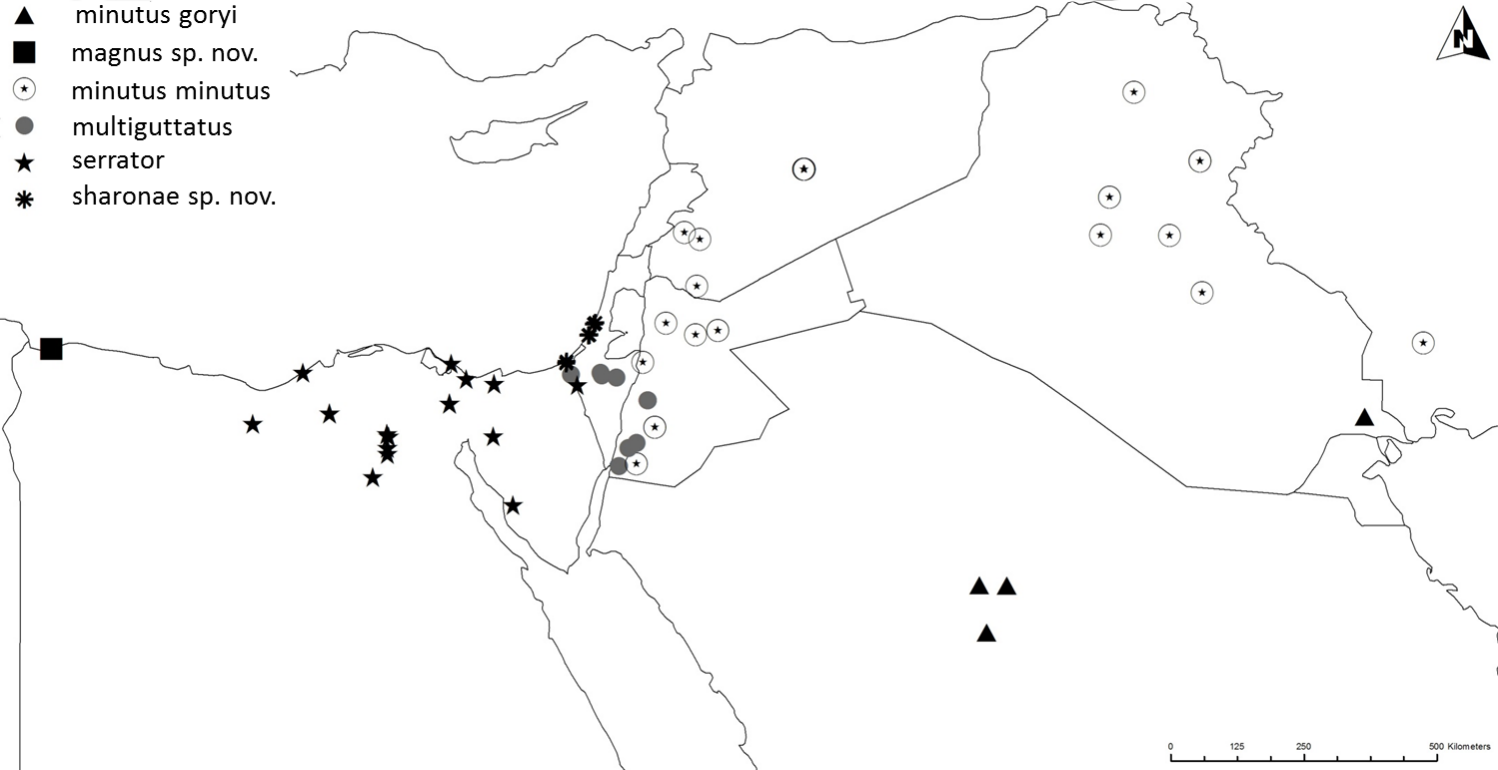
Distributional records of *G. minutus minutus*, *G. minutus goryi*, *G. magnus* sp. n., *G. multiguttatus*, *G. serrator*, and *G. sharonae* sp. n.

##### Conservation.

Unknown.

##### Comments.

Guérin-Méneville described *G. valdanii* as a new species in 1859. Chaudoir (1870: 296), having seen the types that had been collected in Algeria and compared them with *G. serrator* from Egypt, contending that they differed in elytral shape and were a local variation of *G. serrator*.

The type of *valdanii* is lost. Our attempts to find any specimen from the typical series in several museums (incl. NHMB) was unsuccessful, as Guèrin's collection was sold and his material appears to be unavailable (Thierry Deuve pers. comm.). Chaudoir (1870) used *G. valdanii*, but Guérin-Méneville introduced the species as *G. valdani* (sic). Since then, Chaudoir’s spelling has appeared in most of the literature that deals with this species.

**Figure 20.**
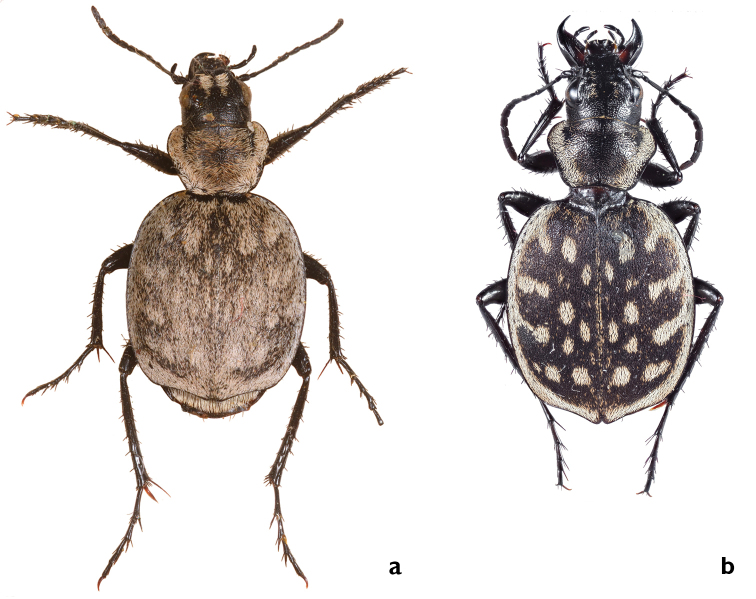
Dorsal habitus of *Graphipterus*: **a** *G. barthelemyi* with greyish scales phase **b** *G. barthelemyi* without grayish scales.

**Figure 21.**
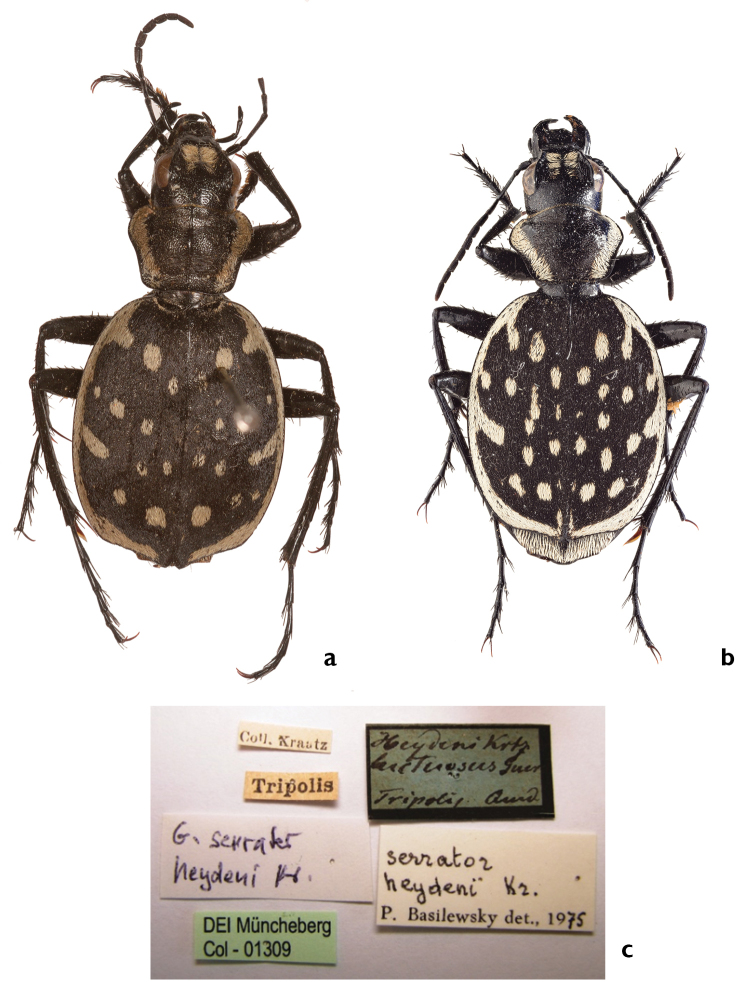
Dorsal habitus of *Graphipterus*: **a** *G. heydeni* **b** *G. luctuosus* **c** *G. heydeni* lectotypes' lables (ZSM).

**Figure 22.**
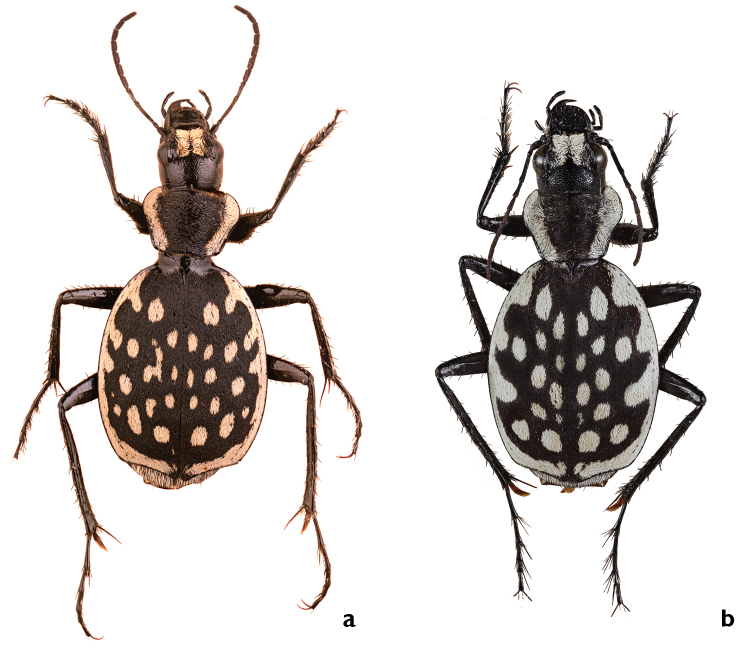
Dorsal habitus of *Graphipterus*: **a** *G. magnus* sp. n. **b** *G. mauretensis* sp. n.

**Figure 23.**
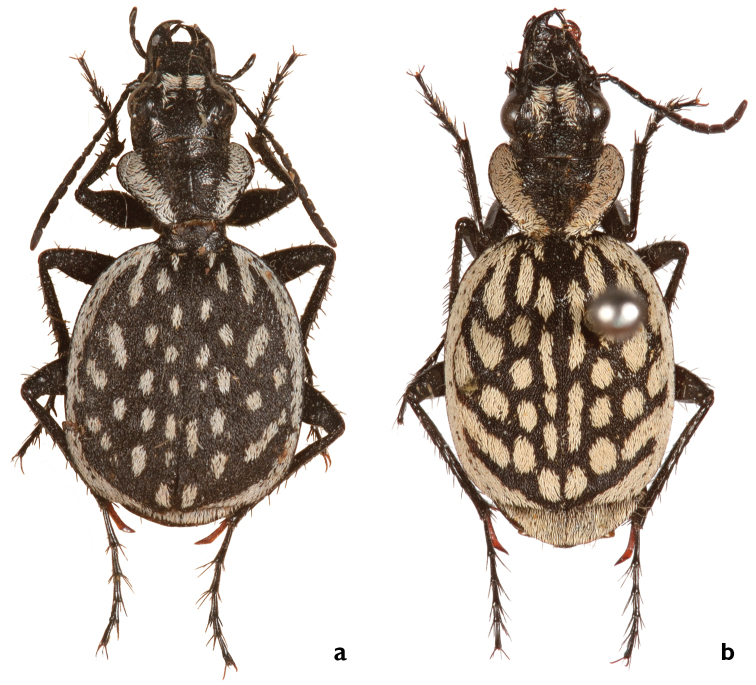
Dorsal habitus of *Graphipterus*: **a** *G. minutus minutus* **b** *G. minutus goryi*.

**Figure 24.**
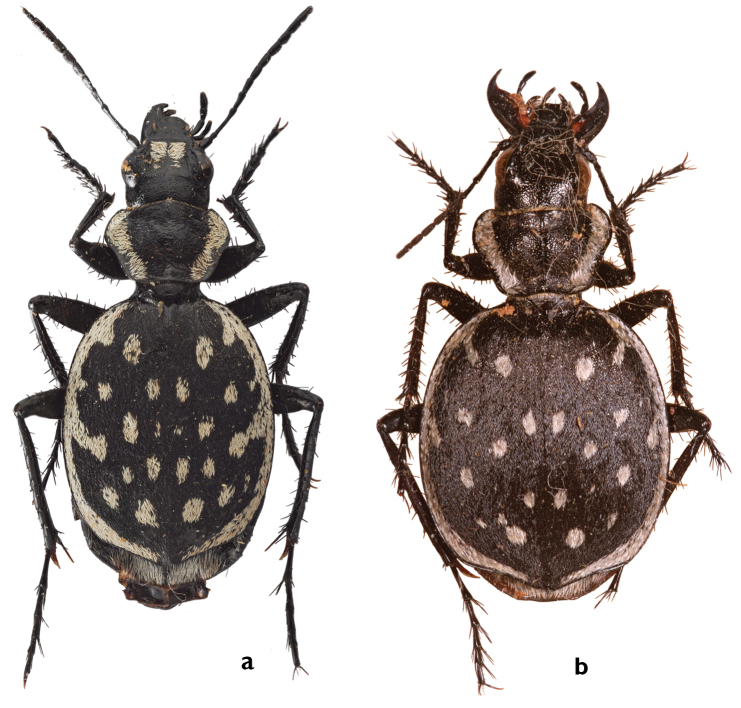
Dorsal habitus of *Graphipterus* **a** *G. multiguttatus* **b.** *G. peletieri*.

**Figure 25.**
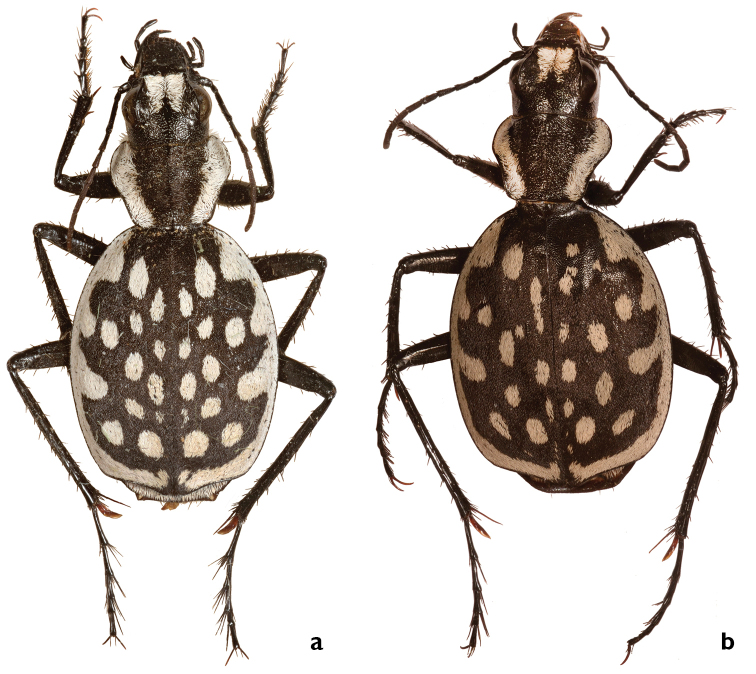
Dorsal habitus of *Graphipterus*: **a** *G. piniamitaii* sp. n. **b** *G. reymondi*.

**Figure 26.**
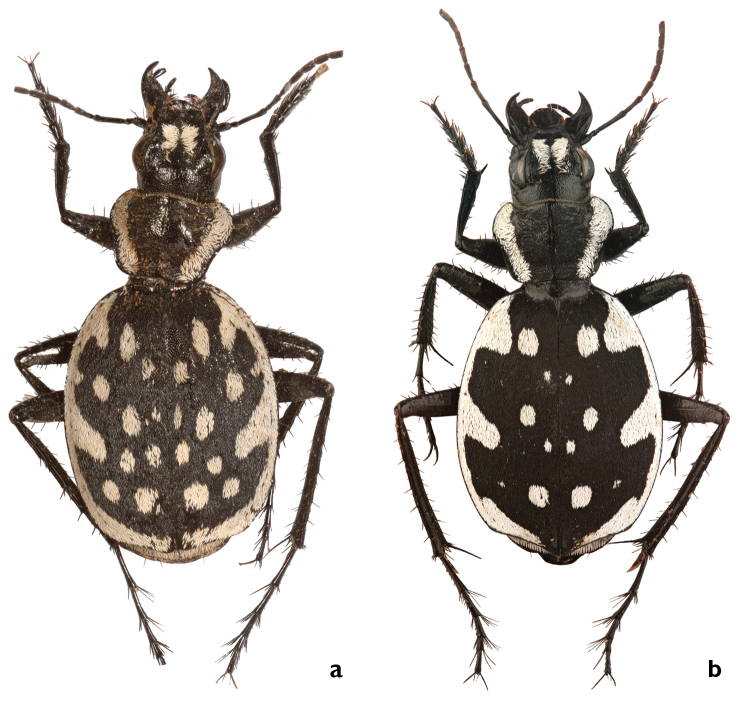
Dorsal habitus of *Graphipterus*: **a** *G. rotundatus* **b** *G. serrator*.

**Figure 27.**
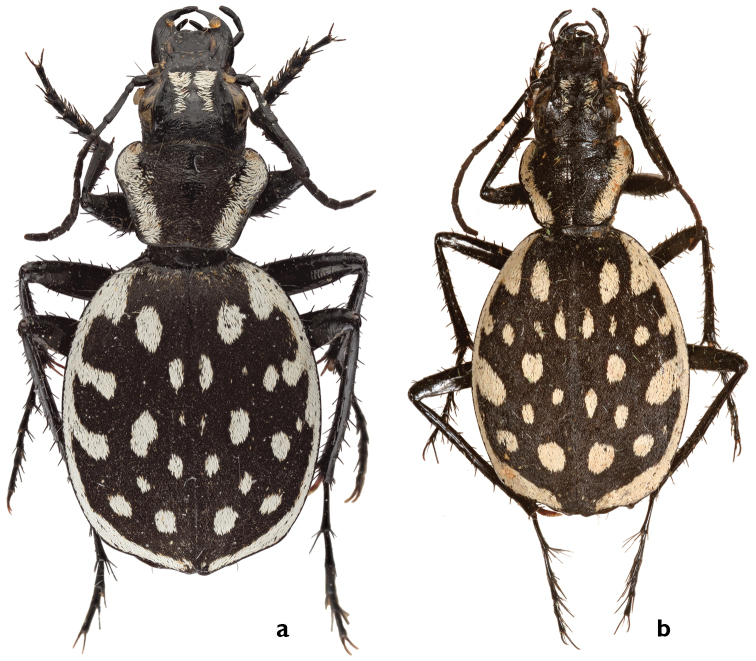
Dorsal habitus of *Graphipterus*: **a** *G. sharonae* sp. n. **b** *G. stagonopsis* sp. n.

**Figure 28.**
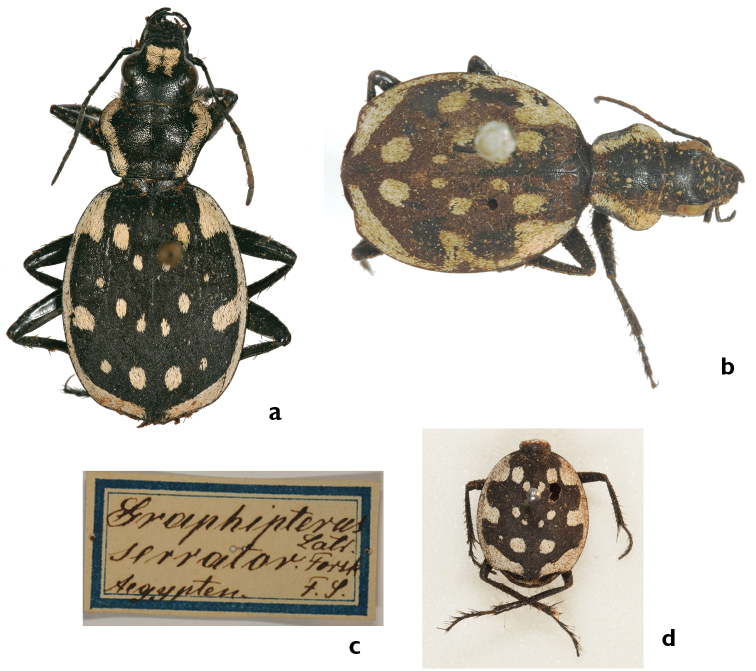
Dorsal habitus of *Graphipterus*: ***a*** *G. valdanii* **b** *G. serrator* holotype (ZMUC) **c** *G. serrator* holotypes’ label **d** *Graphipterus variegatus* holotype (ZMUC).

### Identification key

**Table:** 

1	Stridulatory structure (ventrolaterally on elytral margin and carina on inner side of metafemur) present; pronotum posteriorly concave; median lobe of aedeagus with long curved tip or short, not curved tip (Fig. 9a–f, i–p)	**2**
–	Stridulatory structure absent; pronotum posteromedially not concave; median lobe of aedeagus with wide and flat tip (Fig. 9f, g)	**15**
2	White scales on pronotum restricted to lateral margin (Figs 20–24 except 20a, 20b); elytra and pronotum without grayish or yellowish scales; elytra with white spots and extensions well contrasted to the dark background	**3**
–	Pronotum with white scales extending medially, sometimes to median line (Fig. 20a, b); elytra and pronotum often with grayish or yellowish scales; white spots and extensions on elytra not well contrasted to the dark background. Distribution: north-east Tunisia	***G. barthelemyi***
3	Distribution: Egypt and eastwards.	**4**
–	Distribution: Libya and westwards	**7**
4	Lateral margin of elytra with six extensions; anterior and posterior spots not larger than others; suture conspicuous; apical gap at suture smaller than elytral lateral margin	**5**
–	Lateral margin of elytra with four extensions; anterior and posterior spots larger than others; no conspicuous suture; apical gap at suture wider than elytral lateral margin	***G. serrator***
5	Elytra with 20-24 spots; aedeagus long and thin, with slightly bent tip (Fig. 9d). Distribution: west of the Nile (exclusively known from the north-west Egyptian coast, perhaps also in north-east Libya)	***G. magnus* sp. n.**
–	Elytra with 12-18 spots; aedeagus with strongly bent or unbent tip (Fig. 9 except 9d, f, g). Distribution: east of the Nile	**6**
6	Extension I triangular; aedeagus with strongly bent tip (Fig. 9n). Distribution: exclusively in the sandy coastal plain of Israel and north-east Egypt, perhaps also in Gaza Strip	***G. sharonae* sp. n.**
–	Extension I elongated; aedeagus short with unbend tip (Fig. 9h). Distribution: Sinai Peninsula, Israel, and Jordan	***G. multiguttatus***
7	Distribution: Morocco, Mauritania	**8**
–	Distribution: Algeria, Tunisia, Libya	**9**
8	Mentum with three teeth (Fig. 3d); elytra wider at rear; humeri strongly narrowed; anterior and posterior spots not larger than others; elytra with dark-brown scales; disc of elytra visible between the scales; aedeagus with bent tip	***G. reymondi***
–	Mentum with two teeth (Fig. 3b); elytra relatively elongated oval; humeri slightly narrowed; anterior and posterior spots larger than others; elytra with black scales; disc of elytra not visible between scales; aedeagus short, with unbent tip (Fig. 9e)	***G. mauretensis* sp. n.**
9	Elytra with dark brown scales, disc of elytra visible between them (Fig. 6b); elytral extension I elongated	**10**
–	Elytra with black scales, disc of elytra not visible between them (Fig. 6a); elytral extension I triangular	**11**
10	Three marginal extensions; series of 8-12 elongated spots along suture, forming a broken line; suture conspicuous; apex gap at suture thinner than elytral lateral margin; aedeagus with bent tip (Fig. 9d)	***G. luctuosus***
–	Two marginal extensions; small isolating spots scattered on disc, generally a black beetle; suture not conspicuous; apical gap at suture wider than elytral lateral margin; aedeagus short unbent tip (Fig. 9i)	***G. peletieri***
11	Elytra widest at the posterior third of the elytra, drop-like shape, humeri narrowed; apical sinuation slightly developed	***G. stagonopsis* sp. n.**
–	The widest horizontal line of the elytra is at the middle of the elytra, creates an orb form, humeri rounded; subapical sinuation well developed	**12**
12	Anterior and posterior spots larger than others; apical gap at suture wider than elytral lateral margin; suture not conspicuous	**13**
–	Only posterior spots larger than others; apical gap at suture thinner than elytral lateral margin; suture conspicuous	**14**
13	Elytra with 18–26 spots; mentum with two teeth with concavity between them (Fig. 3b); frontal ridge absent. Distribution: vicinity of Tripoli, Libya	***G. heydeni***
–	Elytra with 10–16 spots; mentum with two teeth as margin between them slightly convex in middle (Fig. 3c); frontal ridge slightly developed. Distribution: Algeria	***G. valdanii***
14	Elytra with 24 spots; most spots wider than lateral margin; lateral cross section quite flat. Distribution: central Tunisia, from the vicinity of Kebili to Gabès	***G. piniamitaii* sp. n.**
–	Elytra with 16–22 (usually 18) spots; most spots thinner than lateral margin; lateral cross section convex. Distribution: Algeria, Tunisia and the coastal region of west Libya	***G. rotundatus***
15	Elytra with 36–40, mostly rounded white spots, including a series of 10–14 round spots along median suture; lateral margin of elytra with two extensions. Distribution: Syria, Jordan, Saudi Arabia, Iraq and western Iran	***G. minutus minutus***
–	Elytra with approx. 30, mostly elongated white spots, usually with several spots fused with lateral margin, and with a series of 10 elongated spots, usually fused to each other along median suture; lateral margin of elytra with six extensions. Distribution: Iraq and Iran	***G. minutus goryi***

## Discussion

### Species delimitation

The *Graphipterus serrator* group shows a high divergence that is exceeded by only a few other species groups of the genus *Graphipterus* (e.g., *G. sennariensis* group, Lorenz 2005). The results of the present study now re-divide the previously “polytypic species” *Graphipterus serrator* from comprising one species with six subspecies, as classified by Basilewsky (1977), the author of the last revision of this genus, into 14 species. Some of Basilewsky’s subspecies have been accepted by Lorenz (2005) and Huber and Marggi (2017) as species. However, these authors did not show any methodological procedure for their decisions (cf. Assmann et al. 2008) and they still accept *Graphipterus serrator* as one species with an extraordinarily large distribution range from Morocco to the southern Levant. The strong increase in species number suggests that the overall number of all insect species, especially beetles, is still underestimated, also in the western Palaearctic. This finding corresponds with other recent findings regarding beetle diversity: e.g., the remarkable increase in beetle species numbers from the western Palaearctic demonstrated by Hendrich et al. (2015), who used DNA barcoding and found numerous overlooked species, even in Central Europe. A macroecological approach suggested that many species from certain parts of the Palaearctic region have been overlooked and an underestimated species number is also assumed for less studied regions (see Schuldt et al. 2009).

Furthermore, the remarkable re-division since the time of Basilewsky (1977) from one species to 14 includes eight re-rankings of historically described species while only five taxa are new to science. Although the total species number of this species group seems to be high, it should be taken into account that the authors do not know of any other wingless Palaearctic ground beetle species that covers a distribution range with a linear expansion of at least ca. 5,500 km, as does *G. serrator* (sensu lato) according to Basilewsky (1977), Lorenz (2005) and Huber and Marggi (2017). In general, the distribution range of flightless ground beetle species is much smaller (cf. Homburg et al. 2013, Homburg et al. 2014). Nearly two-thirds of the western Palaearctic carabid species are endemics, with distribution ranges smaller than ca. 6 × 105 km^2^ (Schuldt and Assmann 2009). Almost all are flightless as the species of the *Graphipterus serrator* group. The distribution ranges of the species as classified after our numerical taxonomic approach fall mostly into the range indicated by Schuldt and Assmann (2009).

One of the weaknesses of both classical and modern taxonomy lies in the definition of an objective decision by which to delimit species, although there seems to be a common ground across many species concepts as to what a species means (Hey 2006). In some cases an increase of species numbers occurs due to the application of different species concepts, as recently discussed for several mammalian taxa (e.g., Zachos 2013). This so-called taxonomic inflation is also known from ground beetles, especially in those cases where allopatric taxa have been elevated from subspecies to species level without the provision of any new findings or a discussion of the reasons for these decisions (Assmann et al. 2008). However, the re-division of the *Graphipterus serrator* group is the only reasonable response to Basilewsky’s previous “lumping” approach. Our decisions were based on a consistent consequence of the application of our threshold value derived from the number of diagnostic characters of sympatrically occurring taxa (Table 1). This approach constitutes an objective method for species delimitation decisions; and one that we propose also be applied for further taxonomic analyses with both sympatric and allopatric taxa.

The decision not to give a different “weight” to pattern and morphological diagnostic characters was based on the problem of defining the “exact right weight”. However, it is important to emphasize the characteristics of the pairs with the lowest diagnostic characters in the matrix: the sympatric pair *luctuosus* and *peletieri* which has been defined as the threshold for delimiting a “good” species, are diagnostic with six characters, five of which are pattern characters, but the shape of the median lobe of aedeagus differs in both species. Another pair with a low diagnostic character number is the allopatric species *serrator* and *valdanii*, also with six characters. However, four of these characters are morphological. The taxa *minutus* and *goryi* are ranked as subspecies as they differ from one another by only four diagnostic characters. No sympatric *Graphipterus* species pair of the *serrator* group is known to have so small morphological and coloration differentiation.

### Endangered taxa in the *Graphipterus serrator* group

Our approach revealed several threatened species, both sympatric and allopatric. Some of them show very small distribution ranges and their habitats have undergone strong losses and fragmentation, such as those of the coastal dune habitats in the southern Levant. For those taxa, the relevant countries, as for example Israel, have special responsibility for their protection as the taxa do not occur anywhere else than in the given country. The coastal plain in Israel, for example, is inhabited by 30 endemic and 118 red list species of vascular plants (Shmida et al. 2011). *Graphipterus sharonae* sp. n., along with the weevil *Achradidius ochraceus* (Tournier, 1874) are the only insect species that have been studied and classified as endemics of the given region, but future studies will probably find additional ones (Friedman pers. comm.).

## Supplementary Material

XML Treatment for Graphipterus

XML Treatment for Graphipterus barthelemyi

XML Treatment for Graphipterus heydeni

XML Treatment for Graphipterus luctuosus

XML Treatment for Graphipterus magnus

XML Treatment for Graphipterus mauretensis

XML Treatment for Graphipterus minutus minutus

XML Treatment for Graphipterus minutus goryi

XML Treatment for Graphipterus multiguttatus

XML Treatment for Graphipterus peletieri

XML Treatment for Graphipterus piniamitaii

XML Treatment for Graphipterus reymondi

XML Treatment for Graphipterus rotundatus

XML Treatment for Graphipterus serrator

XML Treatment for Graphipterus sharonae

XML Treatment for Graphipterus stagonopsis

XML Treatment for Graphipterus valdanii

## Supplementary materials

Supplementary material 1 Material examined Data type: specied data This is an open access article distributed under the terms of the Creative Commons Attribution License (CC BY 4.0), which permits unrestricted use, distribution, and reproduction in any medium, provided the original author and source are credited.Ittai Renan, Thorsten Assmann, Amnon Freidberg
